# AI-assisted discovery of high-temperature dielectrics for energy storage

**DOI:** 10.1038/s41467-024-50413-x

**Published:** 2024-07-19

**Authors:** Rishi Gurnani, Stuti Shukla, Deepak Kamal, Chao Wu, Jing Hao, Christopher Kuenneth, Pritish Aklujkar, Ashish Khomane, Robert Daniels, Ajinkya A. Deshmukh, Yang Cao, Gregory Sotzing, Rampi Ramprasad

**Affiliations:** 1 School of Materials Science and Engineering, Georgia Institute of Technology, Atlanta, GA USA; 2 Materials Science Program, Institute of Materials Science, University of Connecticut, Storrs, CT USA; 3 Electrical Insulation Research Center, Institute of Materials Science, University of Connecticut, Storrs, CT USA; 4 Faculty of Engineering Science, University of Bayreuth, Bayreuth, Germany; 5 Polymer Program, Institute of Materials Science, University of Connecticut, Storrs, CT USA; 6 Present Address: Polymer Program, Institute of Materials Science, University of Connecticut, Storrs, 06296 CT USA; 7 Present Address: Department of Electrical Engineering, Tsinghua University, Beijing, China

**Keywords:** Materials for energy and catalysis, Computational science

## Abstract

Electrostatic capacitors play a crucial role as energy storage devices in modern electrical systems. Energy density, the figure of merit for electrostatic capacitors, is primarily determined by the choice of dielectric material. Most industry-grade polymer dielectrics are flexible polyolefins or rigid aromatics, possessing high energy density or high thermal stability, but not both. Here, we employ artificial intelligence (AI), established polymer chemistry, and molecular engineering to discover a suite of dielectrics in the polynorbornene and polyimide families. Many of the discovered dielectrics exhibit high thermal stability and high energy density over a broad temperature range. One such dielectric displays an energy density of 8.3 J cc^−1^ at 200 °C, a value 11 × that of any commercially available polymer dielectric at this temperature. We also evaluate pathways to further enhance the polynorbornene and polyimide families, enabling these capacitors to perform well in demanding applications (e.g., aerospace) while being environmentally sustainable. These findings expand the potential applications of electrostatic capacitors within the 85–200 °C temperature range, at which there is presently no good commercial solution. More broadly, this research demonstrates the impact of AI on chemical structure generation and property prediction, highlighting the potential for materials design advancement beyond electrostatic capacitors.

## Abstract (web summary)

Dielectrics are essential for modern energy storage, but currently have limitations in energy density and thermal stability. Here, the authors discover dielectrics with 11 times the energy density of commercial alternatives at elevated temperatures.

## Introduction

Electrostatic capacitors are critical energy storage components in advanced electrical systems in the defense, aerospace, energy, and transportation sectors. Compared with other, more vigorously discussed, energy storage devices—such as batteries, fuel cells and supercapacitors—electrostatic capacitors offer unparalleled power density (10^7^ W kg^−1^)^1^. This attribute renders electrostatic capacitors particularly advantageous for deployment in diverse fields, including wind pitch control (with maximum temperatures around 125 °C), hybrid, all-electric and rail vehicles (~150 °C), pulsed power systems (~180 °C), aircraft and aircraft launchers (~300 °C), and space exploration (~480 °C)^2^. An existing challenge, however, is to significantly enhance the energy density *U*_e_—particularly at high temperatures—of electrostatic capacitors, thereby unlocking substantial space and weight saving. For example, DC bus capacitors in power inverters of hybrid vehicles can take up more than 23% of the total weight and 35% of the overall volume^3^. Cooling systems, essential to maintain the stability of temperature-intolerant dielectrics, take up additional space and volume.

Today’s high-power capacitors use biaxially oriented polypropylene (BOPP) as the dielectric, a material that has served well for over three decades and one on which the community, industry, and supply chain have heavily invested. BOPP and similar polyolefins possess low dielectric loss and a large electronic band gap *E*_g_ due, in part, to the absence of *π*-stacking moieties. However, these polymers also have a low dielectric constant *ϵ* and poor mechanical stability at elevated temperatures, both attributes correlated to the lack of *π*-*π* stacking. The interplay of these various factors results in a situation where BOPP maintains sufficient *U*_e_ at room temperature but then rapidly degrades with increasing temperature.

Commercial alternatives to BOPP with high thermal stability have been explored. However, these polymers trade stability for low *E*_g_ and, consequently, a low *U*_e_. Examples of these polymers are polyimide (PI) or Kapton®), polyethertherketone (PEEK), polyetherimide (PEI), and fluorene polyester (FPE). Additional features are expected of these materials, as discussed in subsequent sections, exposing the tightrope that must be walked to craft functional polymer dielectrics that can meet the demands of modern and future technologies.

To a large extent, the function of a polymer is governed by its underlying chemistry. The number of variations that can be produced by chemical permutations in a single polymer is staggering. For instance, derivatizing styrene alone through arene functionalization can lead to 10^7^–10^14^ possibilities (see Section S1). Within the vast expanse of chemical possibilities for all polymers, it is likely that a wide variety of high-performance dielectrics await discovery. Well-trained and calibrated artificial intelligence (AI), capable of handling large numbers that challenge human imagination, can help converge on extraordinary materials rapidly. During the past decade, this powerful approach has guided the discovery, chemical synthesis, and physical characterization of materials across domains^4–6^, including polymer dielectrics with high energy density up to 100 °C^7,8^.

**Fig. 1:**
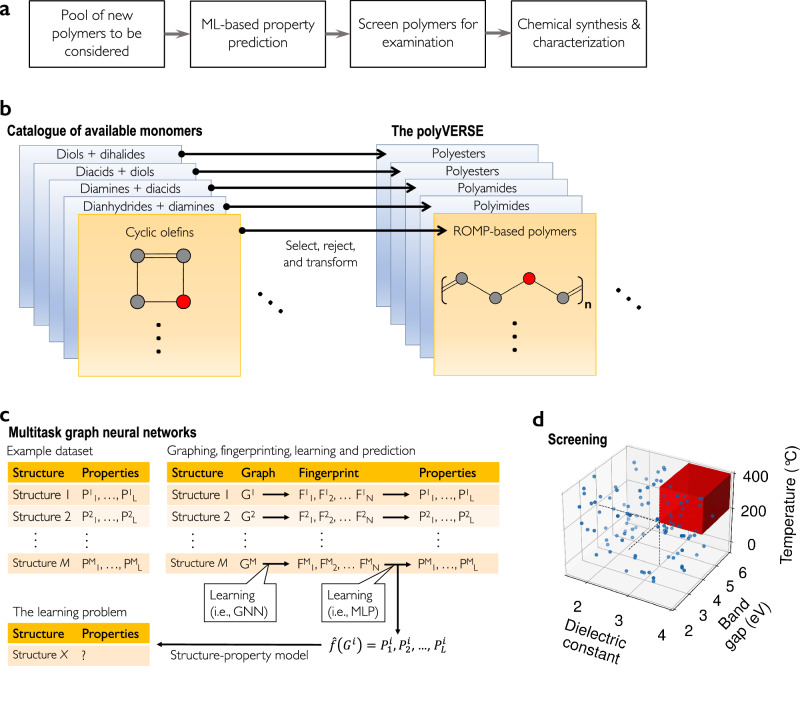
AI-assisted design of polymers for energy storage. **a** Our four-step design approach. First, generate a pool of chemical structures. Then, predict the properties of each. Next, use the predicted properties to screen for the best candidates. Finally, synthesize and characterize the selected candidates. **b** Chemical structures are generated in three steps. First, curate a database of available monomers. Then, choose a reaction. Use that reaction to select/reject each monomer. Finally, the selected monomers are chemically-transformed from a monomer into a polymer repeat unit. **c** Structure-property models are trained using multitask graph neural networks^33^. The starting point is an example dataset containing labeled pairs of the form [Structure, Properties]. Then, each chemical structure is converted to a machine-readable graph, with heavy atoms as nodes and covalent bonds as edges. A model is trained on the data to establish a mapping between a structure’s graph and each property. An intermediate output, also learned during training, is the fingerprint. Properties are predicted for each chemical structure in the polyVERSE database. **d** Screening is performed using a sequence of carefully chosen, application-specific, filters: high glass-transition temperature, band gap and dielectric constant. In the figure, GNN stands for graph neural network and MLP stands for multi-layer perceptron.

Efficient advancement in materials discovery involves selecting or generating a chemical subspace, estimating the properties of each material within it, and then selecting—based at least in part on estimated properties—candidates to synthesize and test. The challenge lies in (1) creating a subspace that is expansive enough to uncover interesting unknown materials while (2) constraining false positives, defined as challenging-to-synthesize hypothetical materials. Additionally, property estimation must be both (3) accurate and (4) efficient, with the latter becoming increasingly crucial as the chemical subspace expands. Addressing all these aspects simultaneously is a non-trivial task. This study introduces the polyVERSE ("polymers designed by Virtually-Executed Rule-Based Synthesis Experiments”) paradigm (Fig. 1a), showcasing its success in achieving these four attributes in the context of high-temperature dielectric search. In this AI-driven approach, polymers are generated from commercially-available monomers using an expert system (Fig. 1b) and properties are estimated using multitask graph neural networks (Fig. 1c). These property estimations are used to guide the selection (screening) of promising polymers from the larger population (Fig. 1d).

**Fig. 2:**
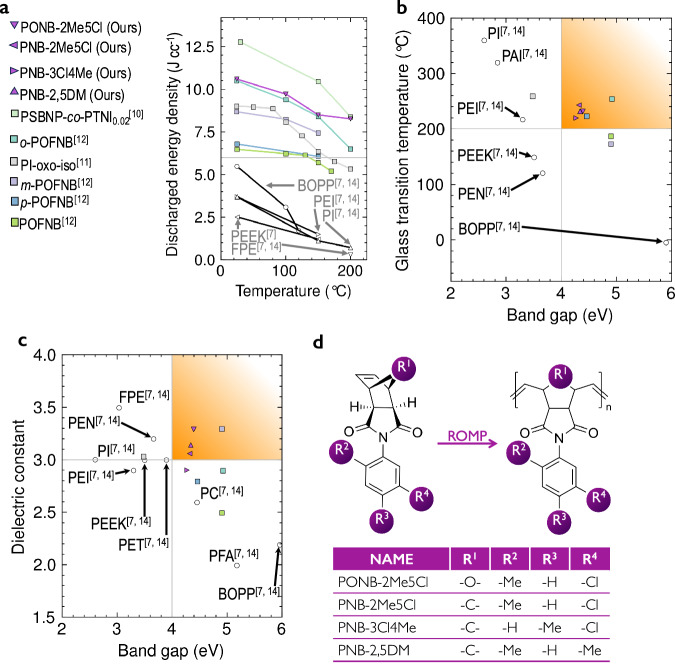
Filling the dielectric void. **a** We find that PONB-2Me5Cl surpasses current state-of-the-art commercial dielectric materials, especially at elevated temperatures. **b**, **c** The high performance of this polymer is related to an improved combination of glass transition temperature, band gap and dielectric constant compared to commercial polymers. **d** The chemical structure of PONB-2Me5Cl and three other polymers discovered in this work. The orange shaded areas in (**b**) and (**c**) denote the desired property value pairs for polymer dielectrics. The glass-transition temperature, band gap, and dielectric constant of all polymers (whether commercial or discovered in this work) included in (**b**) and (**c**) are characterized using the same method in the same lab^7,34^. In the figure, ROMP stands for Ring Opening Metathesis Polymerization. Source data are provided as a Source Data file.

Here, we report a previously unknown polynorbornene dielectric, named PONB-2Me5Cl (see Fig. 2d), with high *U*_e_ over a broad range of temperatures. At 200 °C, as shown in Fig. 2a, the polymer has a *U*_e_ of 8.3 J cc^−1^. This value is over an order of magnitude higher than that of any commercial alternative and places it among the best polymer dielectrics^9–11^ ever reported at this temperature. Below 200 °C, PONB-2Me5Cl also exhibits a high energy density, surpassing all commercial polymers and trailing only PSBNP-co-PTNI0.02^9^. It is worth noting that, for PSBNP-co-PTNI0.02, more steps may be involved (compared to PONB-2Me5Cl) for its synthesis, due to it being a copolymer, and due to the complexity of its comonomers. It is also likely that there may be differences in the measurement protocols used in the previous study relative to the present one^12^.

PONB-2Me5Cl was discovered in silico using polyVERSE, and then subsequently synthesized and characterized. Discovery of the high-performing PONB-2Me5Cl polymer and the development of the polyVERSE paradigm are two outcomes of this work. In addition, we propose a host of polymer design advancements to be considered in the future. These include modified versions of PONB-2Me5Cl (achieved through R-group engineering or the incorporation of nanofillers or coatings) and selected polyimides, a class we prioritize based on existing functional high-temperature polymers^13,14^. These designs display the potential for boosted high-temperature *U*_e_ and reduced loss, while also allowing for synthesis using green solvents.

Using just one of many polymerization templates, the present work has shown the power of AI in producing state-of-the-art polymer dielectrics with remarkable energy storage. Moving on to hundreds of other known templates, it is fair to assume that even better performing materials await discovery.

## Results

### AI-assisted dielectric design

For use in high-temperature applications, the dielectrics in capacitors must be stable at high operating temperatures (e.g., they must have a high glass-transition temperature *T*_g_) while simultaneously exhibiting a high *U*_e_. The maximum *U*_e_ of an electrostatic capacitor using a linear dielectric is determined by the dielectric’s breakdown field *E*_bd_ and its *ϵ* (Eq. (1)).

1 $${U}_{{{{{{{\rm{e}}}}}}}}=\frac{1}{2}{\varepsilon }_{0}\epsilon {E}_{{{{{{{\rm{bd}}}}}}}}^{2}$$

where *ε*_0_ is the vacuum permittivity. Due to the intricate nature of dielectric breakdown mechanisms in polymers, direct simulations of engineering breakdown under realistic conditions are impractical (although the intrinsic breakdown of perfect, defect-free, crystalline systems can be handled^15–17^). A pragmatic alternative approach is to include a positively correlated proxy such as *E*_g_^18^ during the design process. In summary, a polymer with a high *T*_g_ (e.g., >100 K), large *E*_g_ (>4 eV), and high *ϵ* (>3) is likely to exhibit a high *U*_e_ at high temperatures.

Regrettably, the simultaneous achievement of all three property requirements is beyond the capabilities of available polymers. We cast the search for suitable dielectrics as an optimization problem over the space of polymer chemical structures. polyVERSE is a three-step formalization of the problem, including chemical structure generation, property prediction, and screening steps. In the first step, polymer structures are derived from small molecules using established chemical reactions. As a result, the requisite monomer(s) and polymerization reaction for any generated polymer are determined. This advantage is absent from the majority of prior work^19–24^ on polymer chemical structure generation, where reaction chemistry is not explicitly encoded, resulting in a large fraction of structures that lack easily identifiable monomers and reactions.

Recent works, such as the Open Macromolecular Genome (OMG)^25^ and Small Molecules into Polymers (SMiPoly)^26^, have also employed known reactions to generate polymer chemical structures from small molecules. However, it is important to consider certain constraints when dealing with polymer reactions to increase the likelihood of high molecular weight products. Our methodology stands out by addressing these constraints during the structure generation process. Another difference is that, while OMG uses generative modeling to populate their monomer database, we currently rely on commercially available monomers. These pragmatic choices are aimed at increasing the probability of synthetic amenability of the proposed chemical structures.

The starting point of polyVERSE is a set of small molecules, represented by SMILES strings, derived from the databases ZINC15^27^ and ChEMBL^28^. The tranche of small molecules selected from the ZINC15 database included those that were readily purchasable and with standard reactivity. Counterions and chirality were stripped from the SMILES strings. Finally, SAscore^29^ was used to remove complex molecules, whittling the dataset down to ≈8 million unique available monomers.

The next component of the chemical structure generation process is a growing set of handcrafted reaction templates, one per polymerization reaction. Each template contains a set of transformations (i.e., bond-breaking and -forming events) and a set of monomer filters. In AABB-type polycondensation, a polymer is formed through repeated reactions between so-called AA and BB monomers. This reaction involves two monomers, so the template has two distinct monomer filters, each filter containing multiple steps. Figure 1b describes how these elements are used to produce polymers. First, the monomer filters are used to select and reject monomers from the available pool. Then, the selected monomer(s) undergo the transformation(s), forming a product—a polymer repeat unit—that is deposited in the polyVERSE database.

As a first pass, we focus on polymers that can be formed by ring-opening metathesis polymerization (ROMP) since this class contains polymers with the best reported high-temperature *U*_e_ to date^9,11^. The ROMP template contains one chemical transformation, i.e., cleavage at the reactive site’s double bond, depicted in Fig. 2d. The template also contains one monomer filter, since ROMP can proceed using just a single type of cyclic olefin. To begin with, the filter checks if a given molecule is a cyclic olefin. The remaining steps in the filter are motivated by our structure-property models. For simplicity, the models assume perfectly repeating polymers with infinite molecular weight. Because chemical reactions are inherently stochastic, perfect recurrence of the repeating unit along each polymer chain is not always practical. However, this approximation is realistic for polymers with narrow reaction pathways. Thus, our filter only accepts molecules with one cyclic olefin group. Although infinite molecular weight is also not possible in practice, the approximation becomes more apt as the molecular weight of a polymer increases. Thus, our filter only accepts molecules with olefin-containing rings of 3–5 atoms or 7–11 atoms. Rings of 6 or 12+ atoms usually are not sufficiently strained to drive the ROMP reaction to high conversion^30^, resulting in low molecular weight chains. Additionally, molecules with strong electrophilic groups (e.g., halides, acyl halides, carbonyls, and carboxyls) adjacent to the cyclic olefin group are discarded due to their tendency to reduce or distribute the ring strain^31^.

Now that the discussion on chemical structure generation is complete, another crucial component of polyVERSE is described, namely, the mechanism used to model the relationship between structure and properties. In this work, the properties of interest are *T*_g_, *E*_g_, and *ϵ*. Establishing a direct connection between a polymer’s chemical structure and any of these properties is highly nontrivial. Instead, we use data and ML to learn three structure-property models, one each for the aforementioned properties. We note that, while *T*_g_ and *E*_g_ may be modeled with high accuracy using only the chemical structure as input^32,33^, *ϵ* can exhibit a strong dependence on temperature and frequency, in addition to chemistry. Therefore, we focus only on ML predictions of *ϵ* at 100 Hz and room temperature ${\epsilon }_{100}^{{{{{{{\rm{RT}}}}}}}}$ in this work. Each of the three models is trained on relatively large data sets using the Polymer Graph Neural Network (polyGNN) algorithm, as described elsewhere^33^. In polyGNN, a polymer is reduced to its repeat unit. This repeat unit is then converted to a graph with atoms as nodes and chemical bonds as edges. Polymer descriptors are then learned by the algorithm during model training. This attribute makes polyGNN efficient at inference time, because polymer descriptors can be computed on GPUs, rather than on CPUs. On a randomly chosen test set, the trained polyGNN models achieved good accuracy, with a root-mean-square error of 32 °C for *T*_g_, 0.5 for ${\epsilon }_{100}^{{{{{{{\rm{RT}}}}}}}}$, and 0.5 eV for *E*_g_^33^.

In total, 26,858 ROMP-based polymer chemical structures were generated from the set of available monomers using our template. This list was screened for structures with exceptional ML-predicted properties: *T*_g_ > 100 °C, ${\epsilon }_{100}^{{{{{{{\rm{RT}}}}}}}} \, > \, 3$, and *E*_g_ > 4 eV. Next, the remaining structures were rank-ordered according to the product of their predicted properties ${T}_{{{{{{{\rm{g}}}}}}}}\times \,{\epsilon }_{100}^{{{{{{{\rm{RT}}}}}}}}\times \,{E}_{{{{{{{\rm{g}}}}}}}}$. Finally, five of the top twelve candidate structures (see Section S2) were selected as synthetic targets on the basis of raw materials cost and expert evaluation of synthesizability.

### Energy storage performance

Of the five candidates, high molecular weight samples of PONB-2Me5Cl, PNB-2,5dimethyl (PNB-2,5DM), PNB-2Me5Cl, and PNB-3Cl4Me (see Fig. 2d for the chemical structure of each polymer) were prepared using ROMP and cast into films. Polymerization of the fifth polymer was unsuccessful. The resulting films were characterized with differential scanning calorimetry (DSC) to determine *T*_g_, spectrometry to determine *E*_g_, and spectroscopy to determine *ϵ* as a function of temperature and frequency. In general, there is a close agreement between our ML predictions and experimental measurements (see Section S3). The mean absolute prediction error for the four polymers was 12 °C (or 5% in mean absolute percentage error) for *T*_g_, 0.4 eV (8%) for *E*_g_, and 0.7 (22%) for ${\epsilon }_{100}^{{{{{{{\rm{RT}}}}}}}}$.

**Fig. 3:**
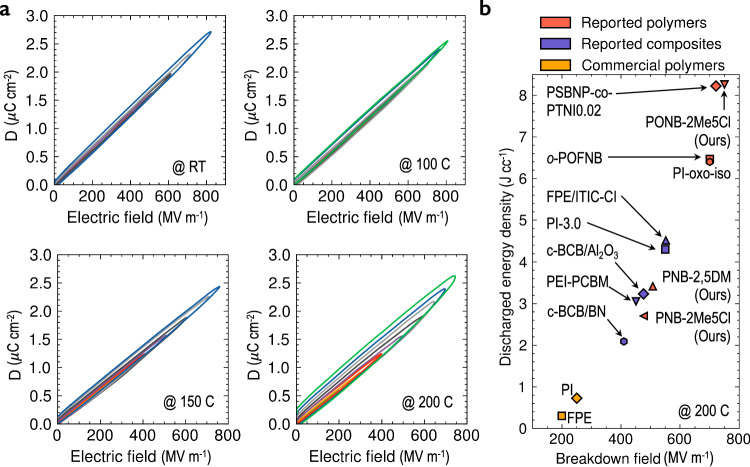
Breakdown field and energy storage performance. **a** Displacement-electric field (*D*–*E*) loops for PONB-2Me5Cl at four temperatures (including room temperature, RT). Line colors differentiate between the electric fields applied in each loop. The field value applied in a particular loop can be ascertained by observing its maximum x-axis value in the plot. **b** Discharged energy density vs. breakdown field at 200 °C for notable commercial and reported polymer-based dielectrics^9–11,35,36,58–60^. Source data are provided as a Source Data file.

**Fig. 4:**
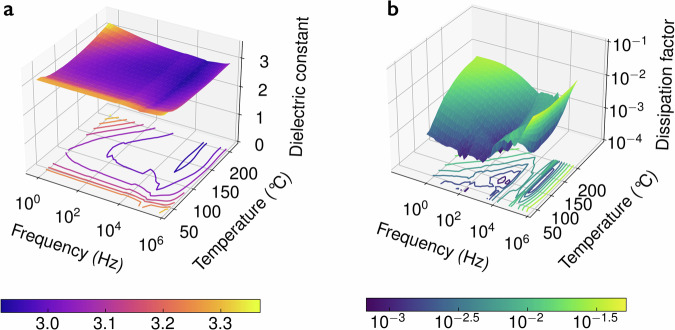
Dielectric constant and dissipation factor. The dielectric constant (**a**) and dissipation factor (**b**) of PONB-2Me5Cl as a function of frequency and temperature. Contour plots are displayed on the bottom wall of each diagram. Source data are provided as a Source Data file.

As predicted by our ML models and depicted in Fig. 2b, c, each of the discovered dielectrics breaks out of the *T*_g_–*E*_g_ and *ϵ*–*E*_g_ bounds exhibited by commercial polymers. The high *T*_g_ of each dielectric film ensures mechanical robustness at high temperatures. PONB-2Me5Cl has the highest *E*_g_ (4.4 eV) of the group of films. The high *E*_g_ acts as a substantial barrier to electron conduction, leading to an unprecedented *E*_bd_ (>800 MV m^−1^ at 100 °C and 750 MV m^−1^ at 200 °C, see Fig. 3a). This property is complemented by a moderate, thermally-stable dielectric constant (see Fig. 4a). The combination, extraordinary *E*_bd_ and moderate *ϵ*, results in elevated *U*_e_ maintained from room temperature to 200 °C. We emphasize in particular that PONB-2Me5Cl possesses a *U*_e_ of 8.3 J cc^−1^ at 200 °C—a *U*_e_ value that is 11 × that of any commercial polymer at this temperature—potentially eliminating the need to consume energy and space with cooling systems in demanding applications such as wind pitch control, hybrid, electric and rail vehicles, and pulsed power systems.

The performance of PONB-2Me5Cl may be understood by contrasting its chemical structure with that of other polymers. For example, PONB-2Me5Cl possesses bicyclic rings and double bonds that stiffen the backbone, while its pendant phenyl ring impedes rotation around the chain axis. These structural features endow PONB-2Me5Cl with a *T*_g_ significantly higher than that of saturated hydrocarbons like BOPP. Thus, PONB-2Me5Cl remains stable at high temperatures, while BOPP completely breaks down near 125 °C. Second, PONB-2Me5Cl contains several *π* systems per repeat unit while BOPP is completely devoid of such features. As a result, the *E*_g_ of BOPP is over 1.5 eV higher than that of PONB-2Me5Cl. Despite this, BOPP’s *E*_bd_ at room temperature is lower than that of PONB-2Me5Cl (750 MV m^−1^ ^34^ compared with 825 MV m^−1^, as shown in Fig. 3a). This suggests that breakdown in BOPP involves more than just electronic factors; thermal and/or electromechanical effects may also contribute. Third, the presence of *π* systems and polar groups (e.g., C=O), raises the *ϵ* of PONB-2Me5Cl above BOPP while maintaining a reasonable dielectric loss (see Fig. 4b).

Although significant strides have been made that explain the interplay between chemical structure and energy storage performance, this research reveals other unresolved aspects. First, we observe that while PNB-2Me5Cl and PNB-2,5DM differ only in the chemical identity of one aryl substituent R_4_, this slight difference alters *E*_bd_ by ~30 MV m^−1^ and *U*_e_ by about 0.7 J cc^−1^ at 200 °C (Fig. 3b). Even more impactful is the backbone chemistry. PNB-2Me5Cl and PONB-2Me5Cl are identical except for R_1_, yet PONB-2Me5Cl possesses increased *E*_g_ and *ϵ*, leading to a striking escalation at 200 °C in *E*_bd_ and *U*_e_ of almost 300 MV m^−1^ and over 5.5 J cc^−1^, respectively (Fig. 3b).

While not yet fully understood, these subtle chemical tweaks can be utilized to design higher-performing polynorbornenes. For example, a straightforward hypothesis is that PONB-2,5DM—containing oxygen at R_1_ and methyl groups at R_2_ and R_4_—would possess a higher *U*_e_ than any of the polymers studied here. Interestingly, the polyVERSE database currently lacks PONB-2,5DM—despite its chemical similarity to the other polymers—due to the absence of its corresponding monomer in the ZINC database version we downloaded. This omission highlights the usefulness of human ingenuity and supervision in chemical structure generation, even when using current state-of-the-art AI.

### Greener pastures

It is appropriate to evaluate and contextualize the environmental impact of the materials proposed in this work. A few key aspects, including lightweighting and chemical synthesis, are highlighted in this section. A lighter vehicle requires less energy to move, resulting in improved fuel efficiency and lower greenhouse gas emissions. Historically, this objective has driven the adoption of lighter load-bearing materials like aluminum, carbon fiber, and plastics. Additional savings may be realized by economizing the capacitor bank, which can account for over 20% of a vehicle’s weight. For example, the high thermal stability of each dielectric in Fig. 3b eliminates the need for capacitor cooling systems. Among these dielectrics, those with higher *U*_e_ are preferred, as this attribute reduces the amount of capacitor material required to store a fixed amount of energy. Regarding this matter, PONB-2Me5Cl shows a large improvement over commercial polymers (e.g., FPE and PI). Further gains are possible. For instance, in special cases, the *U*_e_ and thermal stability of traditional polymers has been boosted by incorporation of semiconducting nanofillers^35,36^. It is therefore also possible that a nanofilled PONB-2Me5Cl composite may have boosted performance compared to the neat polymer. This type of study is left to future work.

We now shift focus from the environmental impacts of lightweighting to that of chemical synthesis. In this process, each synthesis step usually involves (1) energy inputs, (2) raw materials—some of which may originate from natural resources like petroleum-based feedstocks—and (3) the production of waste, some of which could be hazardous. Therefore, from an ecological perspective, shorter synthetic pathways are often preferred. Relative to PONB-2Me5Cl, fair comparisons are *o*-POFNB^11^ and PSBNP-co-PTNI0.02^9^, two recently-discovered polymers with high *E*_bd_ and *U*_e_ at 200 °C (see Fig. 3b). Each polymer also happens to be synthesized by ROMP. PONB-2Me5Cl is synthesized from starting materials in a three-step procedure, two steps to prepare the monomer and one for polymerization (see Section S4). The number of steps is identical for *o*-POFNB. PSBNP-co-PTNI0.02, meanwhile, is a binary copolymer, synthesized in six steps total. One monomer is synthesized from commercially available starting materials in a four-step procedure and the other in a one-step procedure. In the final step, the two monomers are copolymerized. PI-oxo-iso^10^ is another recently-discovered polymer that displays an energy density near that of PONB-2Me5Cl at 200 °C, however it is unclear how many steps were required to synthesize the polymer and its monomers. Overall, these comparisons suggest that the environmental profile of PONB-2Me5Cl is on par with, or better than, the limited group of known polymers in its class.

Along with short synthetic pathways, raw materials—such as solvents—with low impact are desired. Solvents are used in high volumes, including in the production of synthetic polymers, which amounts to 30 million tons annually^37^. Consequently, there has been a heightened pursuit of polymers compatible with green solvents. The dielectrics discovered in this work were polymerized in dimethylacetamide (DMAc) and tetrahydrofuran (THF)—solvents that are decidedly not green. Traditionally, a solvent is defined as green only if its environmental, health, and safety (EHS) impacts are low and the resources required to produce the solvent are sustainable^38^. The importance of these criteria is well documented. In 1989, as part of the Montreal protocol, carbon tetrachloride was banned for its contribution to ozone layer depletion^39,40^. According to the 2015 World Health Organization IARC evaluations, chloroform and dichloromethane are likely to be carcinogenic to humans^41^. Meanwhile, the current production of many solvents, especially hydrocarbons, is dependent on petroleum derivatives.

In this section, we focus on the two greenest solvents in the ref. ^42^ guide: water and ethanol. Both solvents have a recommended EHS profile and are sustainable, with water being a renewable resource and ethanol being derived from biomass at an industrial scale. While the solubility of *o*-POFNB and PSBNP-co-PTNI0.02 in water and ethanol has not been reported, we find that our polymer, PONB-2Me5Cl, is insoluble in both. Additionally, we found that the polymer is not soluble in 1-butanol, and is only partially soluble in ethyl acetate, the next two greenest solvents in the^42^. guide. Motivated by these findings, we trained a polyGNN model (see Methods for more details) on 26,884 data points to predict the room temperature solubility of a given polymer in 61 solvents, including water and ethanol. Combining this Solvent polyGNN with the previously trained triad of polyGNN models, we screen the polyVERSE for polymers predicted to be soluble in water or ethanol while also maintaining good high-temperature dielectric properties—*T*_g_ > 200°C, ${\epsilon }_{100}^{{{{{{{\rm{RT}}}}}}}} \, > \, 3$, and *E*_g_ > 4 eV.

**Fig. 5:**
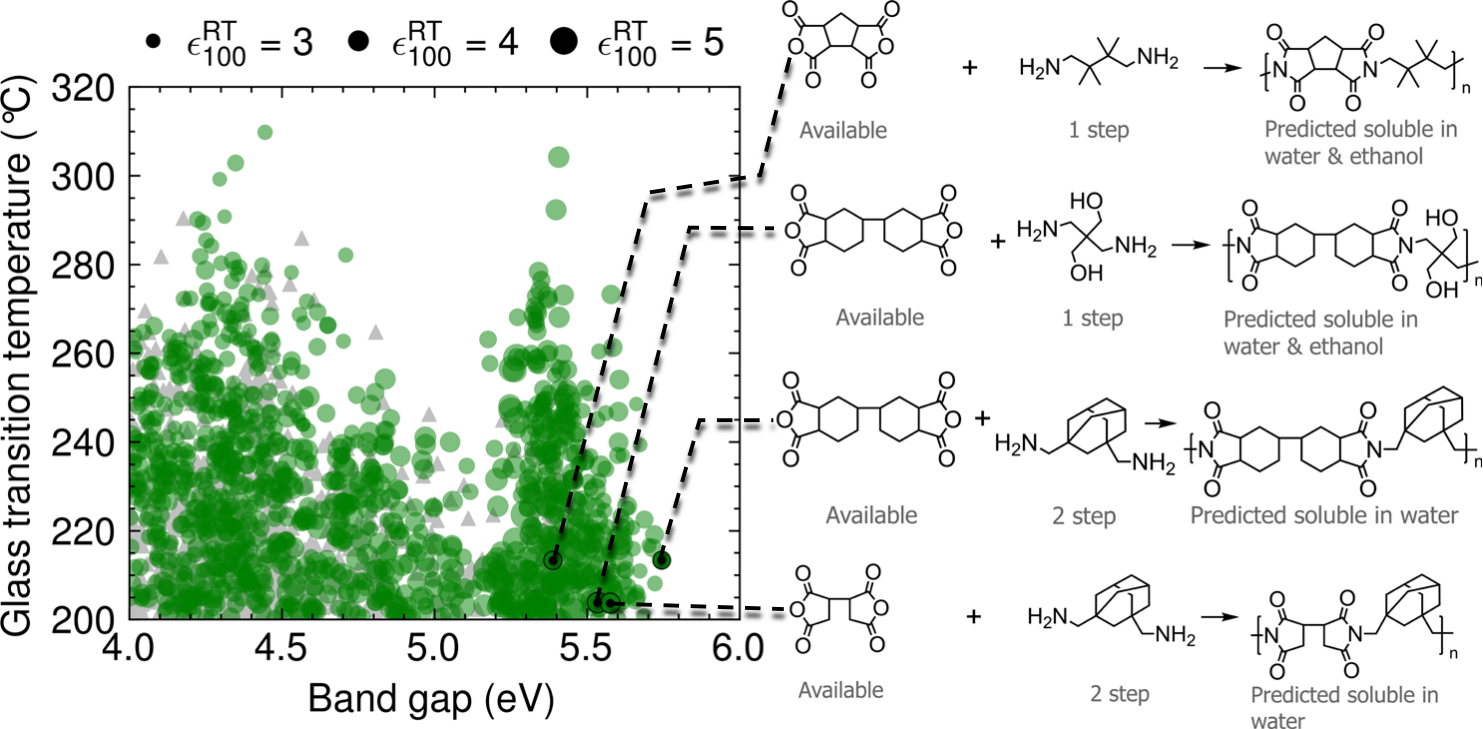
Predicted performance, solubility, and synthetic pathways of suggested polyimides. Out of 66,103 polyimide structures, ~1800 are predicted to exhibit a glass transition temperature > 200 °C, dielectric constant at 100 Hz and room temperature > 3, and band gap > 4 eV. Roughly half of these chemical structures are predicted to be insoluble in water and/or ethanol (gray triangles). The others, represented by green dots, are predicted to be soluble in water and/or ethanol. Four of these structures with feasible synthetic pathways are proposed. “Available” denotes that the molecule is readily available commercially. “*n* step” means the molecule is predicted to be synthesizable in *n* steps from available molecules. Each solubility prediction is performed on the polyamic acid precursor to the polyimide. Source data are provided as a Source Data file.

Our initial polyVERSE database, containing only the ROMP-based structures, yielded zero hits meeting all four criteria. We then turned our attention to the polyimide family, which contains a handful of commercially available polymer dielectrics (e.g., Kapton®, Ultem®, SIXEF-44®, Perflouro polyimide, Upilex-S®) with high *T*_g_^43^. Polyimides are synthesized in two steps. First, the condensation of a dianhyride and diamine yields a polyamic acid prepolymer. Finally, the amic acid groups on the prepolymer are converted to imide groups, commonly by heat treatment. A handful of polyamic acids and polyimides have been reported to be soluble^44–47^ or partially soluble^48^ in water or ethanol. Encouraged by these findings, we crafted a reaction template for polyimides. Applying this template to the data set of available monomers yielded 66,103 candidate polyimide structures, which were then deposited into the polyVERSE database. Out of these, a few hundred chemical structures, depicted as green dots in Fig. 5, were predicted to meet all four criteria. We further selected those polyimides with the most affordable monomers, narrowing the list to four candidate structures. A proposed synthetic route for each polyimide candidate is given at the right of Fig. 5.

It should be noted that the four selected polyimides exhibit slightly lower predicted *T*_g_ values than the four ROMP polymers discovered in this work (204–213 °C vs. 220–243 °C), but significantly higher predicted *E*_g_ values (5.4–5.7 eV vs. 4.3–4.4 eV), which implies higher *E*_bd_ for the polyimides. The high band gap is due, in part, to the absence of aromatic rings in the polyimides. Meanwhile, these polymers can still maintain a relatively high *T*_g_ due to an abundance of aliphatic rings. In fact, previously synthesized fully aliphatic polyimides exhibit *T*_g_ as high as 423 °C^14,49^. We present these polymers to the community in the hope that their promise as high temperature, high energy density polymer dielectrics is evaluated experimentally.

## Discussion

AI, at least in the field of polymer informatics, has truly come of age in the chemical design of materials. This transformation has flourished in the past decade since the Materials Genome Initiative, which has spurred the training of materials scientists, widespread data generation and curation (both computational and experimental), and the proliferation of materials-specific, open-source, informatics software packages.

Building on that foundation, we propose the polyVERSE paradigm for the accelerated intelligent design of polymers rooted in established chemistry. As an initial step, we demonstrate its practical utility for the high-temperature dielectric application, a problem entailing multiple competing material properties. Our approach revealed PONB-2Me5Cl, an exceptional polymer for electrostatic energy storage, especially in high-temperature applications such as wind pitch control, hybrid vehicles and rail, and pulsed power systems. A handful of other prospective dielectrics in the polyVERSE database, including some with green profiles, are recommended. Characterization of these previously unknown polymers has also revealed important chemical design rules for high *U*_e_ at high temperatures. Considering these outcomes, it is clear that the current iteration of polyVERSE has revealed the potential to design synthesizable, functional polymers. We offer this data set^51^ to the community, and wish to note that this data set will continue to grow with new polymers and reaction templates, as they become available in future work.

Looking ahead, though the polymers discovered in this work are promising for a number of applications, there remains a lack of high-performance polymer dielectrics for aircraft and space exploration, which require a high *U*_e_ above 200 °C. A number of strategies should be explored. High-performance fillers or coatings can be added to the PONB-2Me5Cl matrix. Or, by applying the design rules established in this work, the chemical structure of the polymer matrix itself may be slightly tweaked. Likewise, it is fair to assume that a rapid expansion of the polyVERSE database—by adding reaction templates (of which there are likely hundreds), monomer databases, or both—would reveal even better-performing materials.

Any of these approaches to materials discovery would benefit from increased accuracy of the polyGNN models—perhaps using strategies like pretraining^51,52^—to reduce the amount of time spent on bad leads. Moreover, significant resources should be dedicated to a new generation of AI characterized by human interpretability. For example, AI should not merely indicate that substituting a chloroarene with an aryl methyl or replacing a cycloalkane with an alicyclic ether improves the energy storage of a polynorbornene. It should also provide a mechanism, which humans can evaluate and build upon, ultimately leading to a productive human-machine partnership in the quest to understand and harness nature.

## Methods

### Materials

Anhydrous toluene, THF, methanol, ethanol, acetic anhydride, and anhydrous sodium acetate were purchased from Fisher Scientific. Exo-3,6-epoxy-1,2,3,6-tetrahydrophthalic anhydride (Oxanorbornene anhydride), Dichloromethane Anhydrous, 5-cloro-2-methylaniline, and ethyl vinyl ether were purchased from TCI America. cis-5-Norbornene-exo-2,3-dicarboxylic anhydride, 3-chloro-4-methylaniline, and 2.5- dimethylaniline were purchased from Oakwood Chemical. The Grubbs Generation 2 catalyst was used for ring-opening metathesis polymerization and was purchased from Sigma-Aldrich.

### Chemical synthesis

The following monomers were synthesized in this work: ONB-2Me5Cl, NB-Dimethyl, NB-2Me5Cl, and NB-3Cl4Me. Each monomer was synthesized following a similar two-step procedure (Fig. S4). Each reaction was carried out in 500 ml flask under an argon atmosphere. In the first step, 30.5 mmol of the norbornene anhydride with 30 ml of toluene was added to the flask and stirred. Then, the aniline (30.5 mmol) was dissolved in 10 ml toluene and added dropwise to the dispersed solution. The mixture was heated to 40–50 °C for 3 h, then cooled, yielding an amic acid functionalized norbornene.

In the second step, 6–11 g of the amic acid, 0.8–1.8 g sodium acetate, and 5–15 g acetic anhydride (excess) were added to the flask and stirred. The mixture was heated to 60–70 °C for 8 h. The reaction mixture was cooled to room temperature, and a solid precipitate crashed out. The precipitate was filtered, washed, recrystallized, and dried, yielding 3–7 g of pure monomer.

Each monomer was polymerized using ROMP. In a clean and dry flask, 2 g of the monomer was dissolved in 22 ml dichloromethane (DCM) under argon at room temperature. Grubbs 2nd generation catalyst (~22 mg) was dissolved in 4–5 ml of DCM in a vial and added dropwise to the monomer solution. The mixture was stirred for 2 h, quenched using 4–5 ml of ethyl vinyl ether, and then stirred for 20 more min. The solution was then precipitated in cold methanol, purified, and dried, yielding 1.8–1.9 g of pure polymer.

### Material characterization

A Bruker DMX 500 MHz Nuclear Magnetic Resonance (NMR) spectrometer was used to confirm the structure and purity of each monomer and polymer. Thermogravimetric analysis (TGA) and differential scanning calorimetry (DSC) were used for the thermal characterization of synthesized polymers. TGA was performed using a TA Instruments TGA Q-500-0188 at a heating rate of 20 °C min^−1^. DSC was performed using a TA Instruments DSC Q20 with a heating and cooling rate of 10 °C min^−1^. The molecular weight of each polymer was determined using the Waters GPC system, where DMAc was used as the mobile phase and polystyrene standards were used. The band gap of each polymer was calculated by performing UV–Vis spectroscopy on PerkinElmer’s Lambda 1050 UV/Vis/NIR spectrometer.

### Electrical characterization

The dielectric constants and dissipation factors were measured using the commercial dielectric spectroscopy instrument Solartron SI 1260 with the dielectric interface Solartron 1296. Measurement temperature was controlled by placing the sample in the oven Delta Design 9015. A 15 mm gold/palladium film, serving as the electrode, was coated on both sides of each dielectric film. High-field *D*–*E* loop measurements were carried out using a developed Sawyer-Tower polarization loop tester by applying a half-sinusoidal voltage with a frequency of 100 Hz, while measuring the polarization current through the film. The high-voltage amplifier Trek Model 10/40 was used in the system to apply a voltage with a maximum amplitude of 10 kV. For D-E loop measurement, gold/palladium was coated on both sides of the film as electrodes with a diameter of 3 mm by the sputter coating method.

### Generating polyimide structures

polyVERSE polyimides were generated using two monomer filters, and a set of chemical transformations. One filter looked for suitable diamines and the other for suitable dianhydrides. Both filters require the presence of exactly two functional groups, each with similar reactivity to one another. Estimating reactivity is difficult, and so we use the Gasteiger charge^53^ as a proxy. The chemical transformations in this reaction are represented by the following SMARTS patterns: [C:1](=[O:2])[O:3][C:4](=[O:5]).[NH2:6][Ch:7]>>[C:1](=[O:2])[NH:6]([Ch:7])[C:4](=[O:5]) followed by [C:1](=[O:2])[O:3][C:4](=[O:5])>[N:6]>[C:1](=[O:2])[N:6]([C:4](=[O:5]))[#0] followed by [NH2:1][Ch:2]>>[Ch:2][#0]. To minimize the chance of backbiting, we reject proposed polymers in which the shortest path between atoms at each repeat unit edge is fewer than eight atoms.

### Training structure-property models with polyGNN

polyGNN is an approach to training structure-property models. The key elements are multitask learning, neural message passing, and invariance to polymer-specific transformations. By training models to simultaneously learn multiple tasks at once, the risk of generating overfitted predictions for any one specific target property is reduced^54^. As a result, the accuracy of each property is improved. Neural message passing dynamically learns fingerprints during training instead of predefining them. Although an infinite number of features can theoretically be created, the model is incentivized to learn valuable features by minimizing the target property loss through backpropagation. Architectural choices are made to ensure system invariance to translation, addition, and subtraction. These constraints further enhance the quality of learned features. Translation refers to the shift of the periodicity window, resulting in equivalent periodic repeat units such as (-OCC-), (-COC-), and (-CCO-) in polyethylene glycol. Addition (subtraction) involves extending (reducing) a repeat unit by one or more minimal repeat units, as seen in (-COCO-) and (-COCOCO-), which are equivalent repeat units differing only in the addition (or subtraction) of the minimal repeat unit (-CO-).

Using polyGNN, the *T*_g_ of polymers was predicted by a four-task model, with the thermal properties melting temperature, decomposition temperature, and thermal conductivity as the supporting tasks. Training data for the band gap model comes from density functional theory (DFT) calculations on both polymer chain and crystal structures. In this work, *E*_g_ was predicted at the crystal level. In addition to these two tasks, electron affinity and ionization energy were used as supporting tasks. ${\epsilon }_{100}^{{{{{{{\rm{RT}}}}}}}}$ was predicted using a twelve-task model. This model incorporates the following supporting tasks: room-temperature *ϵ* at nine frequencies, DFT-computed zero-frequency *ϵ*, and refractive index from two sources (experiment and DFT). These models are available at https://github.com/Ramprasad-Group/polygnn.

The solubility of dilute polymer-solvent pairs was predicted using a 61-task polyGNN model, one task per solvent. More specifically, the model classifies a polymer-solvent pair as being soluble, partially soluble, or insoluble. The model architecture is identical to the other models, except that the final linear layer outputs a 3-dimensional vector (one dimension per class) instead of a scalar. In addition, a softmax layer is appended to this layer so that the output is in the form of a probability distribution over each class. The weights of this model were optimized using the Adam optimizer and a class-weighted cross-entropy loss function. All weights were initialized according to a Xavier uniform distribution^55^ with a gain of one.

The predictions displayed in Fig. 5 were made using a production version of the solubility model trained on 26,884 data points^56^. The production model is available at https://github.com/Ramprasad-Group/sol_polygnn. Before training the production model, we trained an identical model on two-thirds of the polymer-solvent pairs and tested it on the remaining third. This model achieved an F1-score of 0.724 and an accuracy of 89.7% on all test data (see Section S10 for the confusion matrix and accuracy curves). A subset of the test data is made up of polymers not seen during training. This subset contains 800 polymer-solvent pairs and 587 unique polymers in total. Of these 800 pairs with unseen polymers, 30 have ethanol as the solvent and 29 have water. The model achieved an F1-score of 0.738 and an accuracy of 94.9% on all 800 pairs, an F1-score of 1.0 and an accuracy of 100% on the 30 ethanol pairs, and an F1-score of 0.759 and an accuracy of 75.4% on the 29 water pairs. The last two scenarios, unseen polymer and water or ethanol, represent the primary use case of the model in the wild.

### Supplementary information

Supplementary Information

Peer Review File

### Source data

Source Data

## Data Availability

The polymer chemical structures generated in this work may be found at https://github.com/Ramprasad-Group/polyVERSE/tree/main/Virtual-Polymer/VFS/ROMP_and_polyimide^50^. This represents the first version of the polyVERSE database. The database will grow in future versions, to include polymers and reaction templates beyond those considered in this work.  Source data are provided in this paper.

## Code availability

The code used to generate the polymer chemical structures may be found at https://github.com/Ramprasad-Group/polyVERSE/tree/main/Virtual-Polymer/VFS/ROMP_and_polyimide^50^. The training code and model weights for the polyGNN *T*_g_, *E*_g_, and ${\epsilon }_{100}^{{{{{{{\rm{RT}}}}}}}}$ models may be found at https://github.com/Ramprasad-Group/polygnn^33^. The training code and model weights for the polyGNN solubility model may be found at https://github.com/Ramprasad-Group/sol_polygnn^57^.

## References

[1] Yang, M., Ren, W., Guo, M. & Shen, Y. High-energy-density and high-efficiency polymer dielectrics for high-temperature electrostatic energy storage : a review. *Small* **18**, 2205247 (2022). 10.1002/smll.202205247 36266932

[2] Fan, B. et al. Dielectric materials for high-temperature capacitors. *IET Nanodielectr.* **1**, 32–40 (2018). 10.1049/iet-nde.2018.0002

[3] Chen, Q., Shen, Y., Zhang, S. & Zhang, Q. M. Polymer-based dielectrics with high energy storage density. *Annu. Rev. Mater. Res.* **45**, 433–458 (2015). 10.1146/annurev-matsci-070214-021017

[4] Batra, R., Song, L. & Ramprasad, R. Emerging materials intelligence ecosystems propelled by machine learning. *Nat. Rev. Mater.* 1–24 www.nature.com/natrevmats (2020).

[5] Gómez-Bombarelli, R. et al. Design of efficient molecular organic light-emitting diodes by a high-throughput virtual screening and experimental approach. *Nat. Mater.* **15**, 1120–1127 (2016). 27500805 10.1038/nmat4717

[6] Barnett, J. W. et al. Designing exceptional gas-separation polymer membranes using machine learning. *Sci. Adv.* **6**, eaaz4301 (2020). 32440545 10.1126/sciadv.aaz4301 PMC7228755

[7] Mannodi-Kanakkithodi, A. et al. Rational co-design of polymer dielectrics for energy storage. *Adv. Mater.* **28**, 6277–6291 (2016). 27167752 10.1002/adma.201600377

[8] Treich, G. M. et al. A rational co-design approach to the creation of new dielectric polymers with high energy density. *IEEE Trans. Dielectr. Electr. Insul.* **24**, 732–743 (2017). 10.1109/TDEI.2017.006329

[9] Chen, J. et al. Ladderphane copolymers for high-temperature capacitive energy storage. *Nature* **615**, 62–66 (2023). 36859585 10.1038/s41586-022-05671-4

[10] Wang, R. et al. Designing tailored combinations of structural units in polymer dielectrics for high-temperature capacitive energy storage. *Nat. Commun.* **14**, 1–11 (2023). 37100776 10.1038/s41467-023-38145-w PMC10133333

[11] Deshmukh, A. A. et al. Flexible polyolefin dielectric by strategic design of organic modules for harsh condition electrification. *Energy Environ. Sci.* **15**, 1307–1314 (2022). 10.1039/D1EE02630E

[12] Ladder-like polymer that could halt electrical overheating divides opinion ∣ Research ∣ Chemistry World. https://www.chemistryworld.com/news/ladder-like-polymer-that-could-halt-electrical-overheating-divides-opinion/4017442.article.

[13] Zha, J. W. et al. High-temperature energy storage polyimide dielectric materials: polymer multiple-structure design. *Mater. Today Energy* **31**, 101217 (2023). 10.1016/j.mtener.2022.101217

[14] Zhuang, Y., Seong, J. G. & Lee, Y. M. Polyimides containing aliphatic/alicyclic segments in the main chains. *Prog. Polym. Sci.* **92**, 35–88 (2019). 10.1016/j.progpolymsci.2019.01.004

[15] Sun, Y., Boggs, S. A. & Ramprasad, R. The intrinsic electrical breakdown strength of insulators from first principles. *Appl. Phys. Lett.* **101**, 43 (2012). 10.1063/1.4755841

[16] Sun, Y., Bealing, C., Boggs, S. & Ramprasad, R. 50+ years of intrinsic breakdown. *IEEE Electr. Insul. Mag.* **29**, 8–15 (2013). 10.1109/MEI.2013.6457595

[17] Kim, C., Pilania, G. & Ramprasad, R. From organized high-throughput data to phenomenological theory using machine learning: the example of dielectric breakdown. *Chem. Mater.* **28**, 1304–1311 (2016). 10.1021/acs.chemmater.5b04109

[18] Kamal, D. et al. Computable bulk and interfacial electronic structure features as proxies for dielectric breakdown of polymers. *ACS Appl. Mater. Interfaces* **12**, 37182–37187 (2020). 32705867 10.1021/acsami.0c09555

[19] Ma, R. & Luo, T. PI1M: a benchmark database for polymer informatics. *J. Chem. Inf. Model.* **60**, 4684–4690 (2020). 32986418 10.1021/acs.jcim.0c00726

[20] Batra, R. et al. Polymers for extreme conditions designed using syntax-directed variational autoencoders. *Chem. Mater.* **32**, 10489–10500 (2020). 10.1021/acs.chemmater.0c03332

[21] Liu, D. F., Feng, Q. K., Zhang, Y. X., Zhong, S. L. & Dang, Z. M. Prediction of high-temperature polymer dielectrics using a Bayesian molecular design model. *J. Appl. Phys.* **132**, 014901 (2022). 10.1063/5.0094746

[22] Kim, C., Batra, R., Chen, L., Tran, H. & Ramprasad, R. Polymer design using genetic algorithm and machine learning. *Comput. Mater. Sci.* **186**, 110067 (2021). 10.1016/j.commatsci.2020.110067

[23] Kern, J., Chen, L., Kim, C. & Ramprasad, R. Design of polymers for energy storage capacitors using machine learning and evolutionary algorithms. *J. Mater. Sci.* **56**, 19623–19635 (2021). 10.1007/s10853-021-06520-x

[24] Gurnani, R. et al. polyG2G: a novel machine learning algorithm applied to the generative design of polymer dielectrics. *Chem. Mater.* **33**, 7008–7016 (2021). 10.1021/acs.chemmater.1c02061

[25] Kim, S., Schroeder, C. M. & Jackson, N. E. Open macromolecular genome: generative design of synthetically accessible polymers. *ACS Polym. Au* **3**, 318–330 (2023). 37576712 10.1021/acspolymersau.3c00003 PMC10416319

[26] Ohno, M., Hayashi, Y., Zhang, Q., Kaneko, Y. & Yoshida, R. SMiPoly: generation of synthesizable polymer virtual library using rule-based polymerization reactions. *J. Chem. Inf. Model.* **63**, 5539–5548 (2023). 37604495 10.1021/acs.jcim.3c00329 PMC10498440

[27] Sterling, T. & Irwin, J. J. ZINC 15 - ligand discovery for everyone. *J. Chem. Inf. Model.* **55**, 2324–2337 (2015). 26479676 10.1021/acs.jcim.5b00559 PMC4658288

[28] Gaulton, A. et al. The ChEMBL database in 2017. *Nucleic Acids Res.* **45**, D945–D954 (2017). 27899562 10.1093/nar/gkw1074 PMC5210557

[29] Ertl, P. & Schuffenhauer, A. Estimation of synthetic accessibility score of drug-like molecules based on molecular complexity and fragment contributions. *J. Cheminform.* **1**, 1–11 (2009). 20298526 10.1186/1758-2946-1-8 PMC3225829

[30] Odian, G. *Ring-Opening Polymerization* Ch. 7, 544–618 (John Wiley & Sons, Ltd, 2004). https://onlinelibrary.wiley.com/doi/abs/10.1002/047147875X.ch7.

[31] Sutthasupa, S., Shiotsuki, M. & Sanda, F. Recent advances in ring-opening metathesis polymerization, and application to synthesis of functional materials. *Polym. J.* **42**, 905–915 (2010). 10.1038/pj.2010.94

[32] Doan Tran, H. et al. Machine-learning predictions of polymer properties with Polymer Genome. *J. Appl. Phys.* **128**, 171104 (2020). 10.1063/5.0023759

[33] Gurnani, R., Kuenneth, C., Toland, A. & Ramprasad, R. Polymer informatics at scale with multitask graph neural networks. *Chem. Mater.* **35**, 1560–1567 (2023). 36873627 10.1021/acs.chemmater.2c02991 PMC9979603

[34] Sharma, V. et al. Rational design of all organic polymer dielectrics. *Nat. Commun.* **5**, 1–8 (2014). 10.1038/ncomms5845 25229753

[35] Yuan, C. et al. Polymer/molecular semiconductor all-organic composites for high-temperature dielectric energy storage. *Nat. Commun. 2020 11:1* **11**, 1–8 (2020). 10.1038/s41467-020-17760-x PMC7411043 32764558

[36] Zhou, Y., Zhu, Y., Xu, W. & Wang, Q. Molecular trap engineering enables superior high-temperature capacitive energy storage performance in all-organic composite at 200 °C. *Adv. Energy Mater.* **13**, 2203961 (2023). 10.1002/aenm.202203961

[37] Erdmenger, T., Guerrero-Sanchez, C., Vitz, J., Hoogenboom, R. & Schubert, U. S. Recent developments in the utilization of green solvents in polymer chemistry. *Chem. Soc. Rev.* **39**, 3317–3333 (2010). 20601997 10.1039/b909964f

[38] Capello, C., Fischer, U. & Hungerbühler, K. What is a green solvent? A comprehensive framework for the environmental assessment of solvents. *Green. Chem.* **9**, 927–934 (2007). 10.1039/b617536h

[39] 1987 Montreal Protocol on Substances that Deplete the Ozone Layer. https://cil.nus.edu.sg/databasecil/1987-montreal-protocol-on-substances-that-deplete-the-ozone-layer-as-amended-1990-1992-1995-1997-1999-2007-2016/.

[40] Liang, Q. et al. Constraining the carbon tetrachloride (CCl4) budget using its global trend and inter-hemispheric gradient. *Geophys. Res. Lett.* **41**, 5307–5315 (2014). 10.1002/2014GL060754

[41] Agents Classified by the IARC Monographs, Volumes 1-132 - IARC Monographs on the Identification of Carcinogenic Hazards to Humans. https://monographs.iarc.who.int/agents-classified-by-the-iarc/.

[42] Byrne, F. P. et al. Tools and techniques for solvent selection: green solvent selection guides. *Sustain. Chem. Process.* **4**, 1–24 (2016). 10.1186/s40508-016-0051-z

[43] Li, Q. Advanced Dielectric Materials for Electrostatic Capacitors (The Institution of Engineering and Technology, 2020). https://books.google.com/books/about/Advanced_Dielectric_Materials_for_Electr.html?id=extEzQEACAAJ.

[44] Nakagawa, T., Fujiwara, Y. & Minoura, N. Diffusivity and permeability of poly(*α*-amino acid) membranes to gases. *J. Membr. Sci.* **18**, 111–127 (1984). 10.1016/S0376-7388(00)85029-2

[45] Hsu, S. L. C., Chang, K. C., Huang, Y. P. & Tsai, S. J. A novel synthesis method for the preparation of aromatic poly(imide benzoxazole) from trimellitic anhydride chloride and bis(o-aminophenol). *J. Appl. Polym. Sci.* **88**, 2388–2391 (2003). 10.1002/app.11867

[46] Jiang, S., Hou, H., Agarwal, S. & Greiner, A. Polyimide nanofibers by “green" electrospinning via aqueous solution for filtration applications. *ACS Sustain. Chem. Eng.* **4**, 4797–4804 (2016). 10.1021/acssuschemeng.6b01031

[47] Taublaender, M. J., Reiter, M. & Unterlass, M. M. Highly crystalline, nanostructured polyimide microparticles via green and tunable solvothermal polymerization. *Macromolecules* **52**, 6318–6329 (2019). 10.1021/acs.macromol.9b00985

[48] Han, S. H. et al. Thermally rearranged (TR) polybenzoxazole: effects of diverse imidization routes on physical properties and gas transport behaviors. *Macromolecules* **43**, 7657–7667 (2010). 10.1021/ma101549z

[49] Hasegawa, M., Horiuchi, M., Kumakura, K. & Koyama, J. Colorless polyimides with low coefficient of thermal expansion derived from alkyl-substituted cyclobutanetetracarboxylic dianhydrides. *Polym. Int.* **63**, 486–500 (2014). 10.1002/pi.4532

[50] Gurnani, R. et al. ROMP and polyimide polymers generated via Virtual Forward Synthesis (VFS) 10.5281/zenodo.12570926 (2024).

[51] Hu, W. et al. Strategies for pre-training graph neural networks https://arxiv.org/abs/1905.12265v3 (2019).

[52] Kuenneth, C. & Ramprasad, R. polyBERT: a chemical language model to enable fully machine-driven ultrafast polymer informatics. *Nat. Commun.* **14**, 1–11 (2023). 37433807 10.1038/s41467-023-39868-6 PMC10336012

[53] Gasteiger, J. & Marsili, M. Iterative partial equalization of orbital electronegativity-a rapid access to atomic charges. *Tetrahedron* **36**, 3219–3228 (1980). 10.1016/0040-4020(80)80168-2

[54] Caruana, R., Pratt, L. & Thrun, S. Multitask learning. *Mach. Learn.* **28**, 41–75 (1997). 10.1023/A:1007379606734

[55] Glorot, X. & Bengio, Y. Understanding the difficulty of training deep feedforward neural networks. In *Proc.**T**hirteenth International Conference on Artificial Intelligence and Statistics* (eds Teh, Y. W. & Titterington, M.), Vol. 9 of *Proceedings of Machine Learning Research*, 249–256 (PMLR, Chia Laguna Resort, Sardinia, Italy, 2010). https://proceedings.mlr.press/v9/glorot10a.html.

[56] Kern, J., Venkatram, S., Banerjee, M., Brettmann, B. & Ramprasad, R. Solvent selection for polymers enabled by generalized chemical fingerprinting and machine learning. *Phys. Chem. Chem. Phys.* **24**, 26547–26555 (2022). 36314064 10.1039/D2CP03735A

[57] Gurnani, R. & Ramprasad, R. sol-polygnn 10.5281/zenodo.12570874 (2024).

[58] Li, H. et al. Scalable polymer nanocomposites with record high-temperature capacitive performance enabled by rationally designed nanostructured inorganic fillers. *Adv. Mater.* **31**, 1900875 (2019). 10.1002/adma.201900875 30977229

[59] Li, Q. et al. Flexible high-temperature dielectric materials from polymer nanocomposites. *Nature* **523**, 576–579 (2015). 26223625 10.1038/nature14647

[60] Dong, J. et al. Scalable polyimide-organosilicate hybrid films for high-temperature capacitive energy storage. *Adv. Mater.* **35**, 2211487 (2023). 10.1002/adma.202211487 36894169

