# Author Correction: AI-assisted discovery of high-temperature dielectrics for energy storage

**DOI:** 10.1038/s41467-024-51526-z

**Published:** 2024-08-21

**Authors:** Rishi Gurnani, Stuti Shukla, Deepak Kamal, Chao Wu, Jing Hao, Christopher Kuenneth, Pritish Aklujkar, Ashish Khomane, Robert Daniels, Ajinkya A. Deshmukh, Yang Cao, Gregory Sotzing, Rampi Ramprasad

**Affiliations:** 1 School of Materials Science and Engineering, Georgia Institute of Technology, Atlanta, GA USA; 2 Materials Science Program, Institute of Materials Science, University of Connecticut, Storrs, CT USA; 3 Electrical Insulation Research Center, Institute of Materials Science, University of Connecticut, Storrs, CT USA; 4 Faculty of Engineering Science, University of Bayreuth, Bayreuth, Germany; 5 Polymer Program, Institute of Materials Science, University of Connecticut, Storrs, CT USA; 6 Present Address: Polymer Program, Institute of Materials Science, University of Connecticut, Storrs, 06296 CT USA; 7 Present Address: Department of Electrical Engineering, Tsinghua University, Beijing, China

**Keywords:** Materials for energy and catalysis, Computational science

Correction to: *Nature Communications* 10.1038/s41467-024-50413-x, published online 19 July 2024

In this article the affiliation details for Ajinkya A. Deshmukh were incorrectly given as ‘Matmerize Inc., Atlanta, GA, USA’ but should have been ‘Polymer Program, Institute of Materials Science, University of Connecticut, Storrs, 06296, CT, USA’. The original article has been corrected.

