# Supplementary material for: AI-assisted discovery of high-temperature dielectrics for energy storage
Source: Nat Commun. 2024 Jul 19;15:6107. doi: 10.1038/s41467-024-50413-x (PMC11271506; doi:10.1038/s41467-024-50413-x)
Supplement: Supplementary file 1 — Supplementary Information [file 41467_2024_50413_MOESM1_ESM.pdf]

Supplementary Information

AI-Assisted Discovery of High-Temperature Dielectrics for Energy Storage

Rishi Gurnani†[1], Stuti Shukla†[2], Deepak Kamal†[1], Chao Wu†‡[3,4], Jing Hao[3], Christopher Kuenneth[1,5], Pritish Aklujkar[6], Ashish Khomane[2], Robert Daniels[2], Ajinkya A. Deshmukh[6], Yang Cao[3], Gregory Sotzing[2,6], and Rampi Ramprasad‡[1]

[1] School of Materials Science and Engineering, Georgia Institute of Technology, Atlanta, GA 30332, USA
[2] Department of Chemistry, University of Connecticut, Storrs, CT 06269, USA
[3] Electrical Insulation Research Center, Institute of Materials Science, University of Connecticut, Storrs, CT 06269, USA
[4] Department of Electrical Engineering, Tsinghua University, Haidian, Beijing 100084, China
[5] Faculty of Engineering Science, University of Bayreuth, 95447, Bayreuth, Germany
[6] Polymer Program, Institute of Materials Science, University of Connecticut, Storrs, CT 06269, USA

†These authors contributed equally to the work
‡ Corresponding authors: rampi.ramprasad@mse.gatech.edu, wuchaothu@tsinghua.edu.cn

Contents

S1 Counting styrene derivatives **2**

S2 polyVERSE ROMP candidates **3**

S3 Comparison of ML predictions and measurements **3**

S4 Polynorbornene synthesis **3**
S4.1 Overview . . . 3
S4.2 Synthesis of 2-methyl-5-chloro oxanorbornene monomer (ONB-2Me5Cl) . . . 4
S4.3 Synthesis of 2-methyl-5-chloro polyoxanorbornene (PONB-2Me5Cl) . . . 5
S4.4 Synthesis of dimethyl norbornene (NB-Dimethyl) . . . 5
S4.5 Synthesis of dimethyl polynorbornene (PNB-Dimethyl) . . . 5
S4.6 Synthesis of 2-methyl-5-chloro norbornene (NB-2Me5Cl) . . . 10
S4.7 Synthesis of 2-methyl-5-choro polynorbornene (PNB-2Me5Cl) . . . 10
S4.8 Synthesis of 3-chloro-4-methyl norbornene (NB-3Cl4Me) . . . 13
S4.9 Synthesis of 3-chloro-4-methyl polynorbornene (PNB-3Cl4Me) . . . 13

S5 Casting films **16**

S6 Material characterization **16**
S6.1 Gel permeation chromatography (GPC) . . . 16

S7 Thermal characterization **18**
S7.1 Thermal Gravimetric Analysis (TGA) . . . 18
S7.2 Differential Scanning Calorimetry (DSC) . . . 18

S8 Electronic characterization **18**
S8.1 Band gap measurement . . . 18

S9 Proposed polyimide synthesis **19**

S10 Solubility model **19**

S1 Counting styrene derivatives

In this section, we answer the question, given a palette of f functional groups (e.g., $-$OH, $-$F, etc.) to draw from, how many unique styrene derivatives can be made, mathematically? Restricting our attention to tri-substituted derivatives $d^{(3)}$, the answer is:

$$d^{(3)}(f) = 5f^3 + f^2 \tag{1}$$

Proof. Each derivative can either be positionally symmetric about a (see Fig. S1) or not. An example of a positionally symmetric derivative is $(3 : \mathrm{R}_1, 4 : \mathrm{R}_2, 5 : \mathrm{R}_3)$[1], since the positions $3, 4, 5$ are symmetric about a. An example of a positionally asymmetric derivative is $(2 : \mathrm{R}_1, 4 : \mathrm{R}_2, 5 : \mathrm{R}_3)$, since the positions $2, 4, 5$ are asymmetric about a. So, we have:

$$d^{(3)}(f) = d^{\mathrm{sym}}(f) + d^{\mathrm{asym}}(f) \tag{2}$$

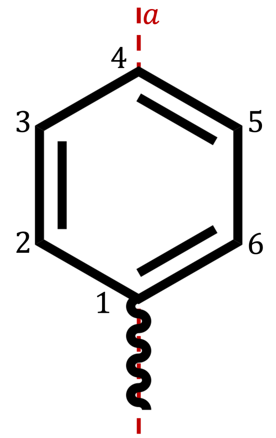

Figure S1: A styrene molecule with the vinyl group (represented by the curly line) at the 1 position. The numbers represent positions on the benzene ring. The dashed line a represents an axis of symmetry.

First, we find an expression for $d^{\mathrm{sym}}(f)$. There are only two combinations of positions on the styrene molecule that are positionally symmetric about a ($C_{\mathrm{sym}} = 2$). These positions are $(3, 4, 5)$ and $(2, 4, 6)$. In either case, the 4 position must take on one of the f groups (therefore, the number of combinations C_4 for the 4 position is f). This leaves two positions left to be filled with groups from f. For these positions, functional group order does not matter, due to positional symmetry about a. For example, $(3 : \mathrm{F}, 4 : \mathrm{F}, 5 : \mathrm{OH})$ is equivalent to $(3 : \mathrm{OH}, 4 : \mathrm{F}, 5 : \mathrm{F})$. Thus, we can use the general equation for number of combinations with replacement C_{R}, which is:

$$C_{\mathrm{R}} = \binom{n + r - 1}{r}$$

where n is the number of objects and r is the number of samples. In our case, $n = f$ and $r = 2$, so we have $C_{\mathrm{R}}(f) = \binom{f+1}{2}$. Putting it all together:

$$d^{\mathrm{sym}}(f) = C_{\mathrm{sym}} \times C_4(f) \times C_{\mathrm{R}}(f) = 2f\binom{f+1}{2} = f^3 + f^2 \tag{3}$$

Next we find an expression for $d^{\mathrm{asym}}(f)$. For positionally asymmetric derivatives, functional group order does matter. For example, $(2 : \mathrm{OH}, 4 : \mathrm{F}, 5 : \mathrm{OH})$ is not equivalent to $(2 : \mathrm{F}, 4 : \mathrm{OH}, 5 : \mathrm{OH})$. There are $\binom{5}{3} = 10$ combinations of functional group positions on styrene. Two of these are positionally symmetric about a, leaving 8 positionally asymmetric combinations. Each of these combinations is equivalent to the combination you get when you flip it over the axis a, leading to 4 unique positionally asymmetric combinations. For example, the combination $(2 : \mathrm{R}_1, 4 : \mathrm{R}_2, 5 : \mathrm{R}_3)$ is equivalent to the combination $(3 : \mathrm{R}_3, 4 : \mathrm{R}_2, 6 : \mathrm{R}_1)$. For each unique combination, the number of functional group permutations is f^3. Therefore:

$$d^{\mathrm{asym}}(f) = 4f^3 \tag{4}$$

Plugging in Eq. 3 and Eq. 4 into Eq. 2 yields Eq. 1. □

We use our data set of purchasable molecules to estimate reasonable values of f. In this data set, there are 56,348 molecules matching the template in Fig S2.

[1] Note that we are referring here to **positional** symmetry about a, not molecular symmetry. Molecular symmetry depends on the identies of $\mathrm{R_i}$. For example, $(3 : \mathrm{F}, 4 : \mathrm{OH}, 5 : \mathrm{F})$ displays molecular symmetry while $(3 : \mathrm{OH}, 4 : \mathrm{OH}, 5 : \mathrm{F})$ displays molecular asymmetry.

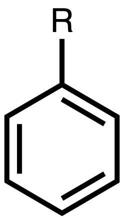

Figure S2: Template for a benzene derivative. R is any group that does not contain an aromatic ring.

Each match contains a unique functional group. The number of such groups without any double/triple bonds is $5,401$. The number of such groups without any rings or double/triple bonds is $2,274$. The number of such groups with four or fewer heavy atoms and no double/triple bonds is 196.

$$d^{(3)}(56348) = 8.95 \times 10^{14}$$
$$d^{(3)}(5401) = 7.88 \times 10^{11}$$
$$d^{(3)}(2274) = 5.88 \times 10^{10}$$
$$d^{(3)}(196) = 3.77 \times 10^{7}$$

Considerations of stereochemistry and mono-, di-, tetra-, or penta-substituted styrene derivatives would further increase the population.

S2 polyVERSE ROMP candidates

Twelve polyVERSE ROMP candidates that were highly ranked by our polyGNN models [1] were considered for synthesis. The top 10 ranked candidates are shown in Fig. S3a. Each of these candidates have monomers that are expensive to purchase. Instead, the monomers would need to be synthesized in-house. As an alternative, we made a separate list of candidates with monomers that could be directly purchased for < \$20 per gram, and then ranked these by polyGNN prediction. The top two candidates are shown in Fig. S3b. In this work, we attempted synthesis of candidates 3, 4, 8, and 9 in Fig. S3a and candidate 2 in Fig. S3b. We were successful with the first four candidates.

S3 Comparison of ML predictions and measurements

Some polyGNN predictions were validated against experimental measurements carried out in this work. These quantities are listed in Table S1.

	E_g, eV	ϵ_{100}^{RT}	T_g, °C
PONB-2Me5Cl	4.39 (4.69)	3.29 (4.02)	232 (214)
PNB-Dimethyl	4.34 (4.76)	3.14 (3.57)	232 (219)
PNB-2Me5Cl	4.32 (4.61)	3.06 (3.80)	243 (236)
PNB-3Cl4Me	4.27 (4.68)	2.90 (3.78)	220 (228)

Table S1: A comparison of measured and predicted values for three properties. Measured values are shown in plain text and predicted values are shown in parentheses.

S4 Polynorbornene synthesis

S4.1 Overview

Monomers were synthesized in a two-step reaction as the scheme shown in Fig. S4. Reactions were carried out in a 500 ml single neck round bottom flask with a magnetic stirrer under an argon atmosphere. For the first step of the reaction, a 1-mole equivalent of norbornene anhydride with toluene was added to the flask and stirred. The 1-mole equivalent of aniline derivatives was dissolved in toluene 0.5-mole equivalent of a mole of limiting reagent) and added dropwise to the dispersed solution. The mixture was heated to 40–50 °C for 3 hrs and then cooled down to give a white precipitate of amic acid. The precipitate was then filtered and dried under a vacuum to give amic acid.

In the second step, a 1-mole equivalent of the amic acid from the first step, 0.5-mole equivalent of sodium acetate and 2–3 mole equivalent of acetic anhydride were added to the flask with a magnetic stirrer, and the mixture was then heated to 60–70 °C for 8 hrs. The reaction mixture was cooled down to room temperature, crashing out a solid white precipitate. The precipitate was filtered under vacuum and then washed several times with water by extraction using dichloromethane (DCM). The collected

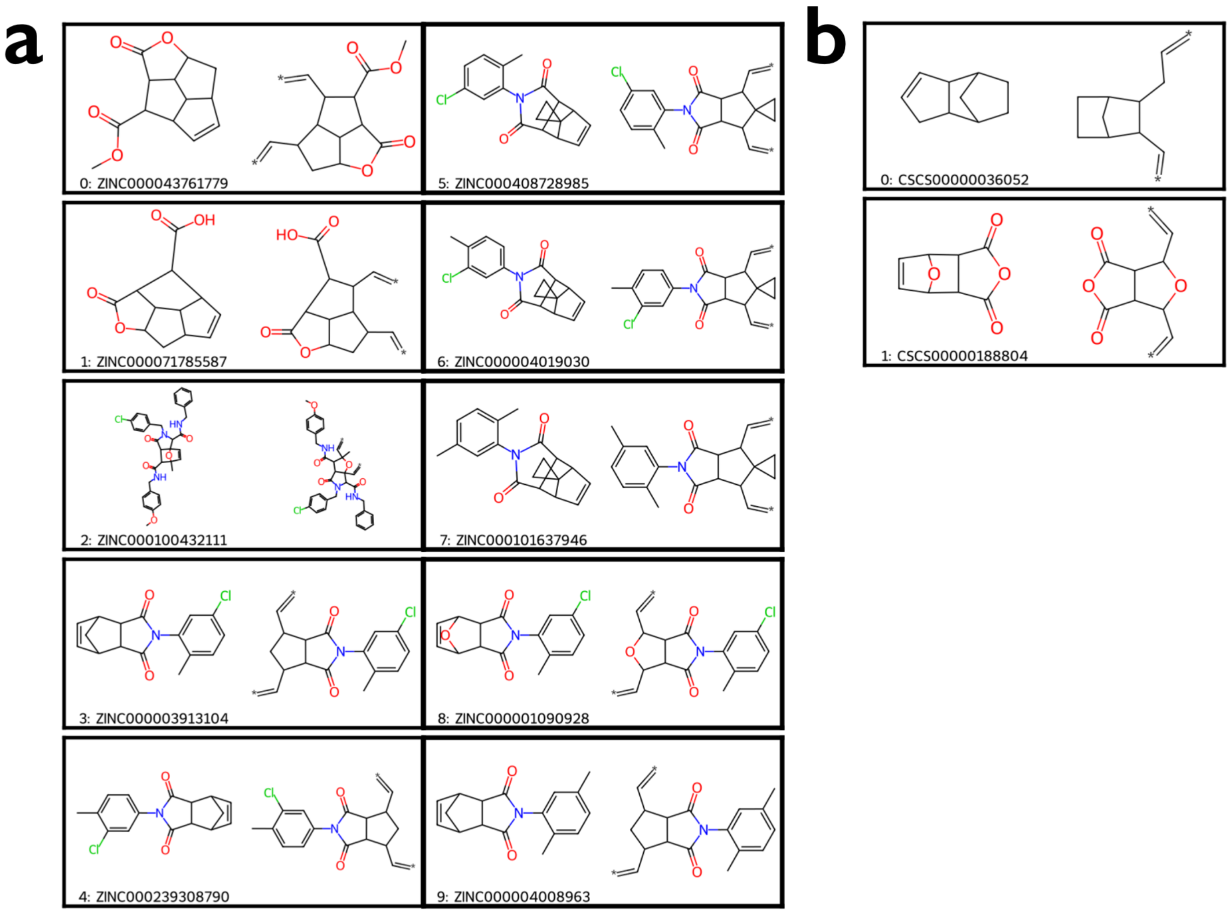

Figure S3: polyVERSE ROMP candidates ranked highly by polyGNN. a) Shows the 10 highest ranking candidates regardless of monomer price. b) Shows the 2 highest ranking candidates with cheap monomers. The left image in each cell shows the monomer and the corresponding ZINC or ChemSpace ID. The right image shows the repeat unit.

organic layer was then collected and evaporated using a rotary evaporator. The solid white product was recrystallized using ethanol and dried under vacuum at 60 °C for 12 hrs to give a pure monomer [2].

The monomers from Fig. S4 were polymerized using ring-opening metathesis polymerization (as shown in Fig. 1d in the main body). In a clean and dry 500 ml single neck round bottom flask, ∼ 2.0 g of monomer was dissolved in 22 ml DCM under argon at room temperature. Grubbs 2nd generation catalyst (∼ 22 mg, 0.026 mmol) was dissolved in 4–5 ml of DCM in a vial and added dropwise to the monomer solution. The reaction mixture was stirred for 2 hrs and then quenched using 4–5 ml of ethyl vinyl ether and stirred for 20 more minutes. The solution was then precipitated in cold methanol to get a white polymer. The obtained polymer was further purified using Soxhlet extraction with methanol for 48 hrs. The polymer was then dried under vacuum at 60 °C for 24 hrs to give 1.8–1.9 g of pure polymer. Similarly, other monomers were also synthesized using the same synthesis scheme and monomer-to-catalyst ratio as a monomer. The details for synthesis and NMR spectra for these monomers and polymers are explained below.

S4.2 Synthesis of 2-methyl-5-chloro oxanorbornene monomer (ONB-2Me5Cl)

The monomer was synthesized in a two-step reaction, shown in Fig. S4. The reaction was carried out in a 500 ml single neck round bottom flask with a magnetic stirrer under an argon atmosphere. For the first step of the reaction, 7.03 g (43.4 mmol) of exo-3,6-epoxy-1,2,3,6-tetrahydrophthalic anhydride with 30 ml of toluene was added to the flask and stirred. 2-methyl-5-chloroaniline 6 g (42.4 mmol) was dissolved in 10 ml toluene and added dropwise to the dispersed solution. The mixture was heated to 40–50 °C for 3 hrs and then cooled down to give a pale-yellow precipitate of amic acid. The precipitate was then filtered and dried under a vacuum to give 10.8 g (35.2 mmol) of amic acid with an ∼ 83% yield.

In the second step, 10.8 g of amic acid from the first step, ∼ 1.74 g sodium acetate, and ∼ 12–15 g acetic anhydride (excess) were added to the flask with a magnetic stirrer, and the mixture was then heated to 60–70 °C for 8hrs. The reaction mixture was cooled down to room temperature, and a solid white precipitate crashed out. The precipitate was filtered under vacuum and then washed several times with water by extraction using dichloromethane (DCM). The collected organic layer was then evaporated using a rotary evaporator. The solid white product was recrystallized using ethanol and dried under vacuum at 75 °C for 12 hrs to give 6.8 g of pure monomer (yield ∼ 66%).

The relevant spectra for ONB-2Me5Cl are given in Figures S5, S6, and S7. In summary, the ^{1}H NMR spectrum of ONB-2Me5Cl (500 MHz, $CDCl_3$) exhibits peaks at δ 7.37-7.13 (3H, multiplet), 6.92-6.62 (2H, doublet), 5.46-5.43 (2H, doublet), 3.09-3.07 (2H, doublet), and 2.17-2.14 (3H, doublet). The ^{13}C NMR spectrum (500 MHz, Chloroform-d) reveals peaks at δ 175.16, 136.82, 132.17, 129.74, 128.11, 81.48, 47.72, and 16.89. Additionally, the mass spectrum shows peaks at m/z 290.0584 $[M+H]^+$, 307.0831 $[M+NH_4]^+$, 312.0316 $[M+Na]^+$, 579.1039 $[2M+H]^+$, 596.0977 $[2M+NH_4]^+$, 601.1033 $[2M+Na]^+$, and 890.1062 $[3M+Na]^+$, with the HRMS m/z calculated for $C_{15}H_{12}ClNO_3$ $[M+H]^+$ being 290.0506, which matches the observed peak at 290.0584.

S4.3 Synthesis of 2-methyl-5-chloro polyoxanorbornene (PONB-2Me5Cl)

The obtained monomer was polymerized using ring-opening metathesis polymerization. In a clean and dry 500 ml single neck round bottom flask, 2 g of ONB-2Me5Cl monomer was dissolved in 22 ml DCM under argon at room temperature. Grubbs 2nd generation catalyst (22 mg, .026 mmol) was dissolved in 4–5 ml of DCM in a vial and added dropwise to the monomer solution. The reaction mixture was stirred for 2 hrs and then quenched using 4–5 ml of ethyl vinyl ether and stirred for 20 more minutes. The solution was then precipitated in cold methanol to get a white polymer. The obtained polymer was further purified using Soxhlet extraction with methanol for 48 hrs. The polymer was then dried under vacuum at 75 °C for 24 hrs to give 1.8 g of pure polymer with 90% yield.

The relevant spectra for PONB-2Me5Cl are given in Figures S8 and S9. In summary, the ^{1}H NMR spectrum of PONB-2Me5Cl (500 MHz, DMSO) exhibits peaks at δ 7.58-7.35 (3H, multiplet), 6.05-5.80 (2H, multiplet), 5.08-4.52 (2H, multiplet), 3.62-3.55 (2H, doublet), and 2.13-1.77 (3H, multiplet). The ^{13}C NMR spectrum (300 MHz, dimethyl sulfoxide-d6) reveals peaks at δ 174.96, 135.37, 132.53, 131.68, 130.74, 129.44, 128.70, 80.37, 77.11, 53.30, and 17.07.

S4.4 Synthesis of dimethyl norbornene (NB-Dimethyl)

The monomer was synthesized in a two-step reaction as in Fig. S4. The reaction was carried out in a 500 ml single neck round bottom flask with a magnetic stirrer under an argon atmosphere. For the first step of the reaction, 5 g ($\sim$ 30.5 mmol) of cis-5-norbornene-exo-2,3-dicarboxylic anhydride with 30 ml of toluene was added to the flask and stirred. 2,5-dimethyl-aniline 3.7 g ($\sim$ 30.5 mmol) was dissolved in 10 ml toluene and added dropwise to the dispersed solution. The mixture was heated to 40–50 °C for 3 hrs and then cooled down to give a pale-yellow precipitate of amic acid. The precipitate was then filtered and dried under a vacuum to give 6.5 g (23 mmol) of amic acid with an $\sim$ 75% yield.

In the second step, 6 g amic acid from the first step, $\sim$ 0.86 g sodium acetate, and $\sim$ 5 g acetic anhydride (excess) were added to the flask with a magnetic stirrer, and the mixture was then heated to 60–70 °C for 8 hrs. The reaction mixture was cooled down to room temperature, and a solid white precipitate crashed out. The precipitate was filtered under the vacuum and then washed several times with water by extraction using dichloromethane (DCM). The collected organic layer was then collected and evaporated using a rotary evaporator. The solid white product was recrystallized using ethanol and dried under vacuum at 75 °C for 12 hrs to give 3.27 g of pure monomer (yield $\sim$ 67%).

The relevant spectra for NB-Dimethyl are given in Figures S10, S11, and S12. In summary, the ^{1}H NMR spectrum of Dimethyl-NB (500 MHz, $CDCl_3$) exhibits peaks at δ 7.26-7.17 (2H, multiplet), 6.92-6.85 (1H, multiplet), 6.40-6.38 (2H, singlet), 3.47-3.46 (2H, singlet), 2.94-2.92 (2H, doublet), 2.37 (3H, singlet), 2.17-2.14 (3H, doublet), and 1.72-1.61 (2H, multiplet). The ^{13}C NMR spectrum (500 MHz, Chloroform-d) reveals peaks at δ 177.51, 138.29, 137.17, 132.84, 131.42, 130.83, 128.81, 128.22, 48.64, 45.73, 43.42, 21.27, and 17.54. Additionally, the mass spectrum shows peaks at m/z 268.1285 $[M+H]^+$, 285.1597 $[M+NH_4]^+$, 290.1166 $[M+Na]^+$, 306.0927 $[M+K]^+$, 535.2548 $[2M+H]^+$, 552.2929 $[2M+NH_4]^+$, and 557.2458 $[2M+Na]^+$, with the HRMS m/z calculated for $C_{17}H_{17}NO_2$ $[M+H]^+$ being 268.1259, which matches the observed peak at 268.1285.

S4.5 Synthesis of dimethyl polynorbornene (PNB-Dimethyl)

The obtained monomer was polymerized using ring-opening metathesis polymerization. In a clean and dry 500 ml single neck round bottom flask, 2 g of NB-dimethyl monomer was dissolved in 22 ml DCM under argon at room temperature. Grubbs 2nd generation catalyst (22.6 mg, $\sim$ 0.027 mmol) was dissolved in 4–5 ml of DCM in a vial and added dropwise to the monomer

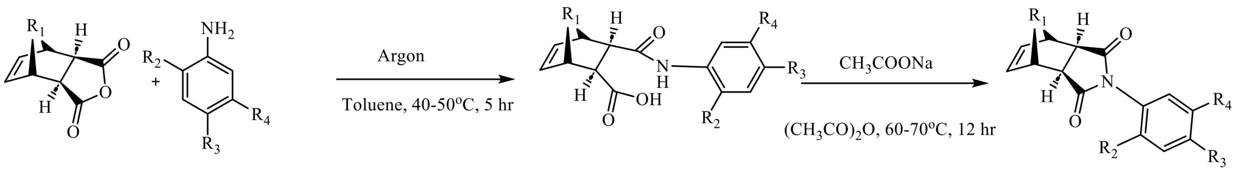

NAME	R_1	R_2	R_3	R_4
ONB-2Me5Cl	O	Me	H	Cl
NB-2Me5Cl	C	Me	H	Cl
NB-3Cl4Me	C	H	Me	Cl
NB-2,5DM	C	Me	H	Me

Figure S4: General scheme of monomer synthesis. "DM" stands for "dimethyl".

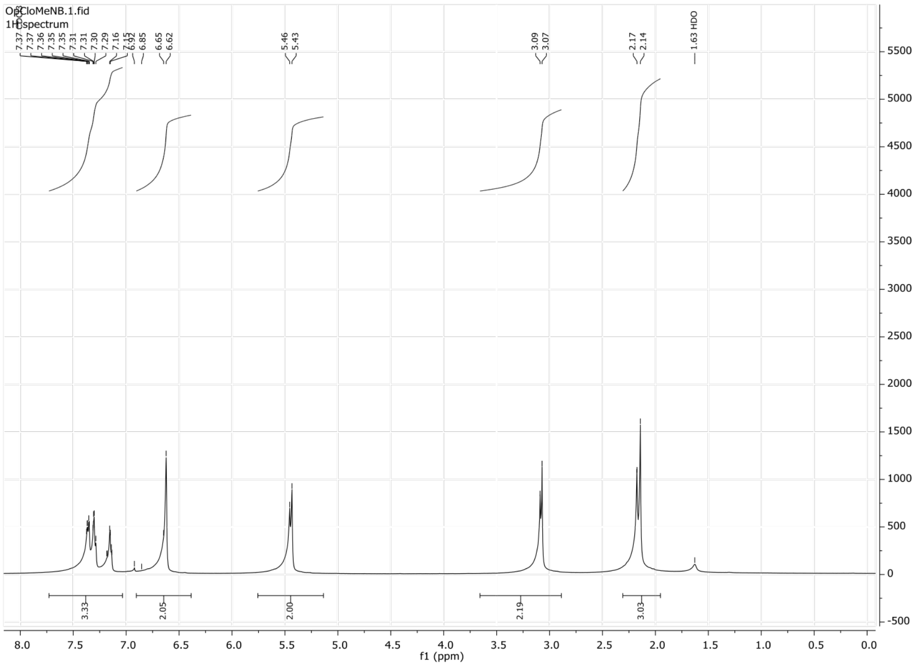

Figure S5: ^{1}H NMR (500 MHz, $CDCl_3$) for ONB-2Me5Cl.

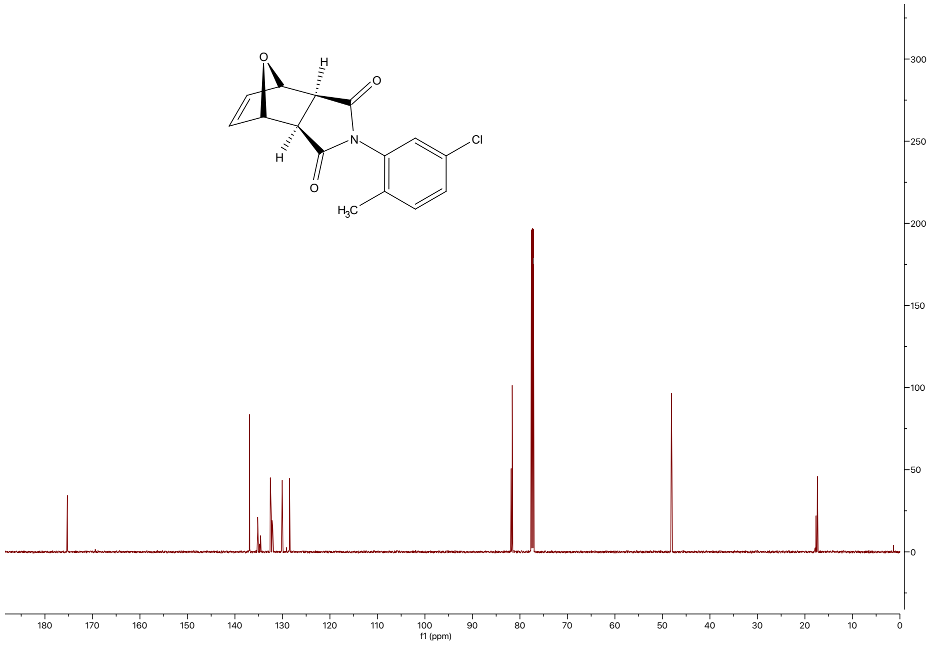

Figure S6: ^{13}C spectrum (500 MHz, $CDCl_3$) for ONB-2Me5Cl.

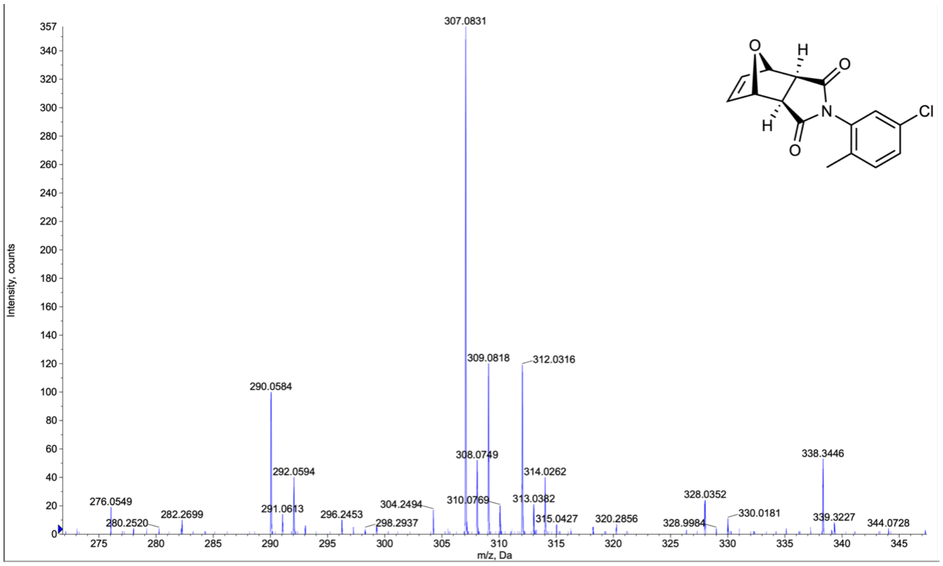

Figure S7: HRMS mass spectrum (positive ion mode) of compound ONB-2Me5Cl ($C_{15}H_{12}ClNO_3$).

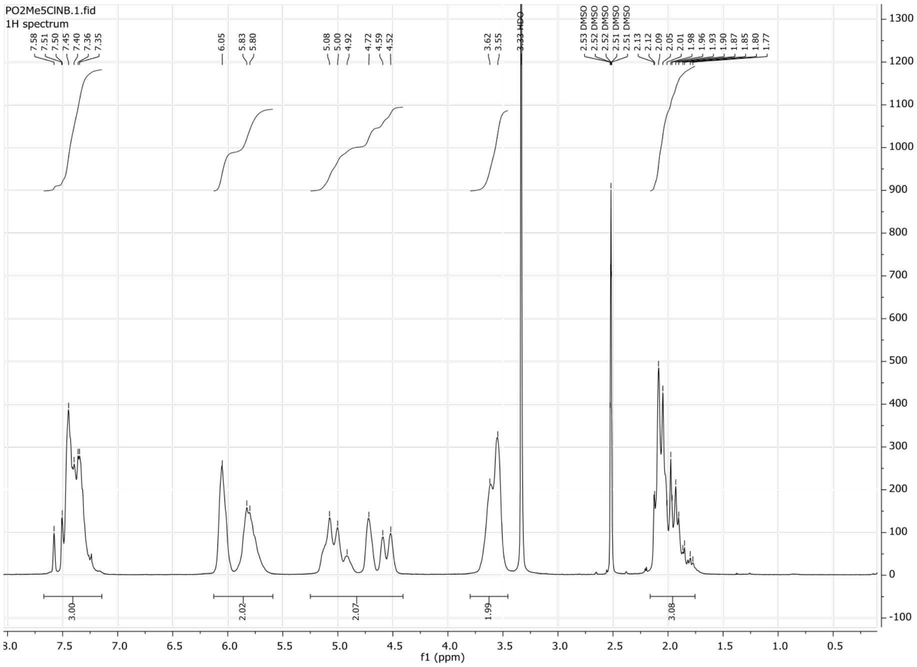

Figure S8: ^{1}H NMR (500 MHz, DMSO) for PONB-2Me5Cl.

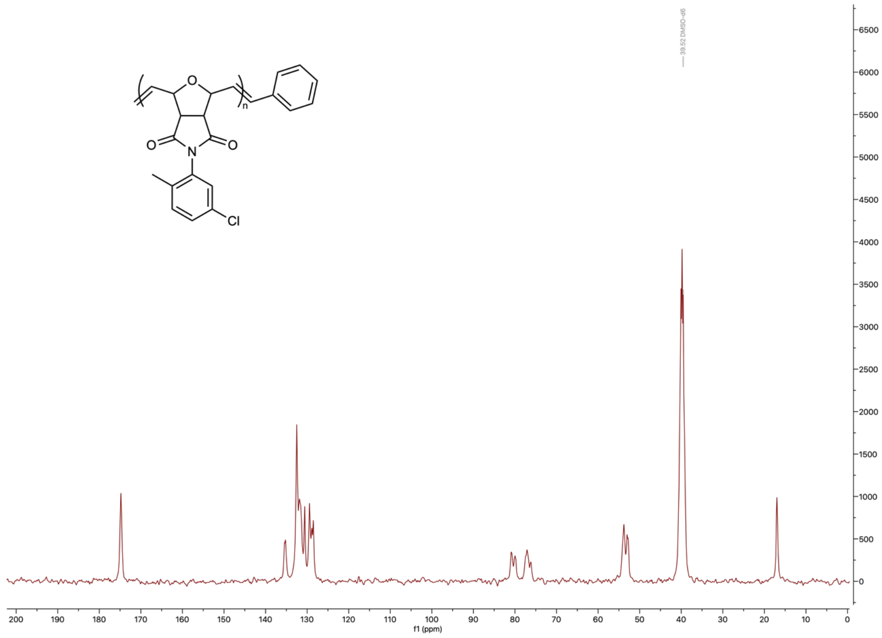

Figure S9: ^{13}C spectrum (300 MHz, DMSO) of PONB-2Me5Cl.

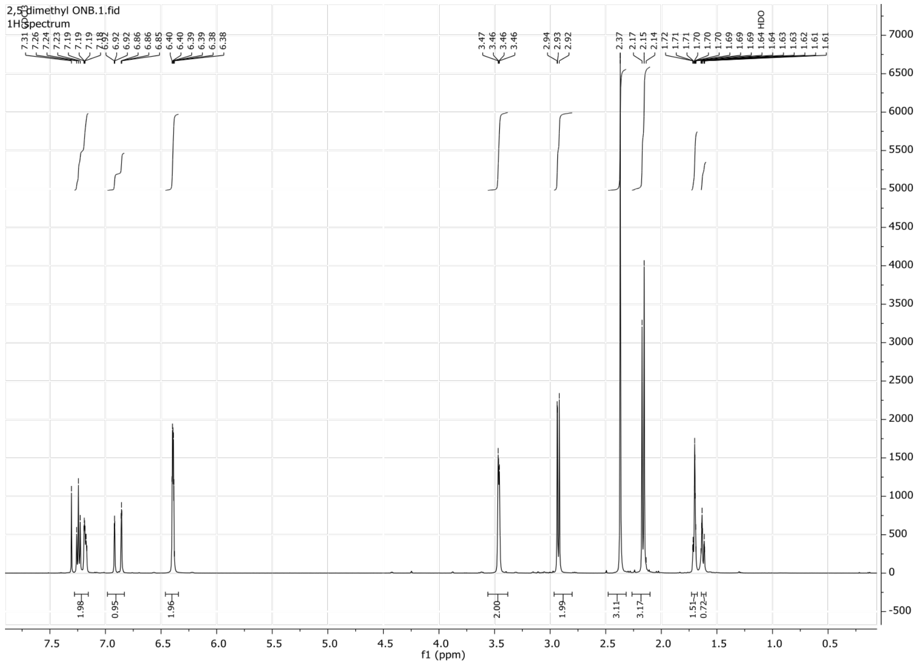

Figure S10: ^{1}H NMR (500 MHz, $CDCl_3$) for Dimethyl-NB.

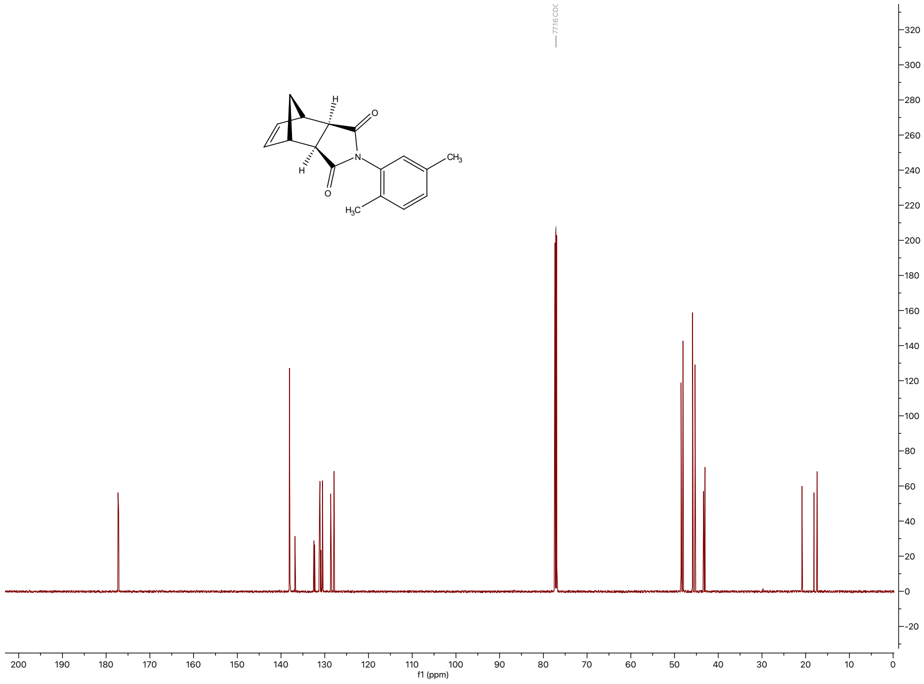

Figure S11: ^{13}C spectrum (500 MHz, $CDCl_3$) of Dimethyl-NB.

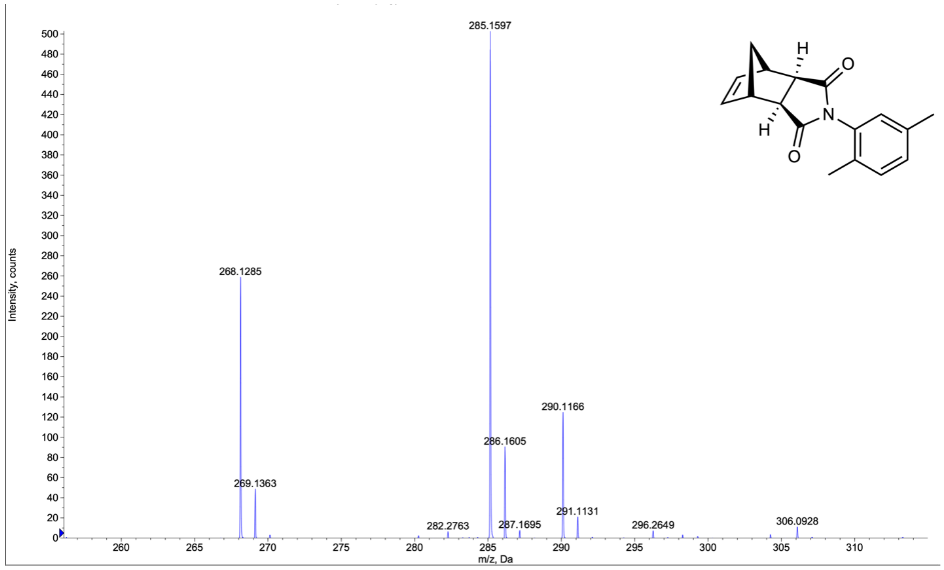

Figure S12: HRMS mass spectrum (positive ion mode) of compound NB-Dimethyl ($C_{17}H_{17}NO_2$).

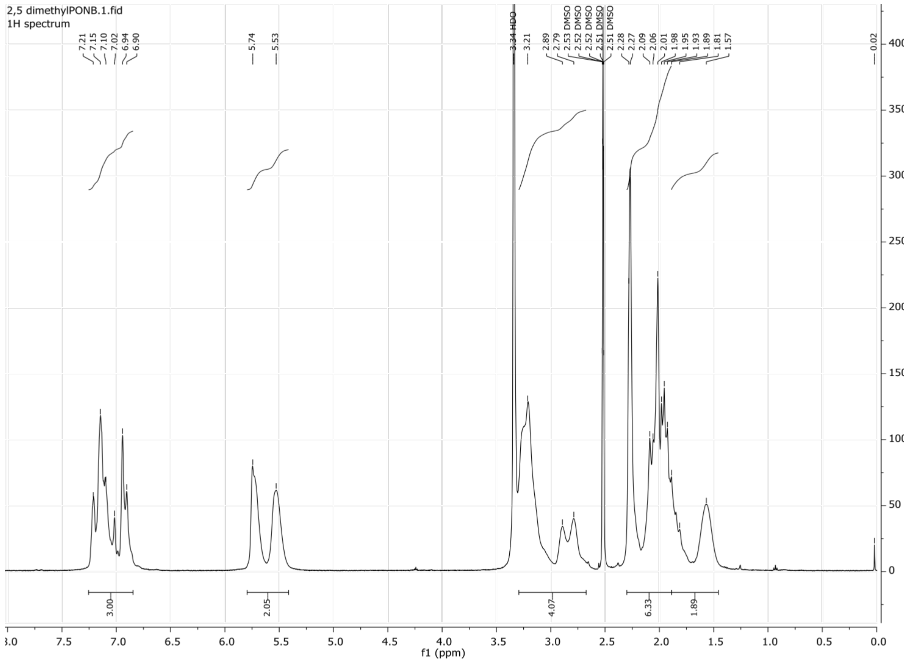

Figure S13: ^{1}H NMR (500 MHz, DMSO) for PNB-Dimethyl.

solution. The reaction mixture was stirred for 2 hrs and then quenched using 4–5 ml of ethyl vinyl ether and stirred for 20 more minutes. The solution was then precipitated in cold methanol to get a white polymer. The obtained polymer was further purified using Soxhlet extraction with methanol for 48 hrs. The polymer was then dried under vacuum at 75 °C for 24 hrs to give 1.9 g of pure polymer with 95% yield.

The relevant spectra for PNB-Dimethyl are given in Figures S13 and S14. In summary, the ^{1}H NMR spectrum of PNB-Dimethyl (500 MHz, DMSO) exhibits peaks at δ 7.21-6.90 (3H, multiplet), 5.74-5.53 (2H, doublet), 3.21-2.79 (4H, multiplet), 2.28-1.81 (6H, multiplet), and 1.57 (2H, singlet). The ^{13}C NMR spectrum (300 MHz, dimethyl sulfoxide-d6) reveals peaks at δ 177.32, 135.78, 132.36, 130.30, 129.55, 128.82, 51.55, 45.27, 42.26, 41.02, 77.11, 20.28, and 16.68.

S4.6 Synthesis of 2-methyl-5-chloro norbornene (NB-2Me5Cl)

The monomer was synthesized in a two-step reaction as in Fig. S4. The reaction was carried out in a 500 ml single neck round bottom flask with a magnetic stirrer under an argon atmosphere. For the first step of the reaction, 5 g (30.5 mmol) of cis-5-norbornene-exo-2,3-dicarboxylic anhydride with 30 ml of toluene was added to the flask and stirred. 2-methyl-5-chloro aniline 4.31 g (30.5 mmol) was dissolved in 10 ml toluene and added dropwise to the dispersed solution. The mixture was heated to 40–50 °C for 3 hrs and then cooled down to give a pale-yellow precipitate of amic acid. The precipitate was then filtered and dried under a vacuum to give 7.7 g (25.2 mmol) of amic acid with an 82.6% yield.

In the second step, 7.7 g amic acid from the first step, $\sim$ 0.94 g sodium acetate, and $\sim$ 5 g acetic anhydride (excess) were added to the flask with a magnetic stirrer, and the mixture was then heated to 60–70 °C for 8 hrs. The reaction mixture was cooled down to room temperature, and a solid white precipitate crashed out. The precipitate was filtered under the vacuum and then washed several times with water by extraction using dichloromethane (DCM). The collected organic layer was then collected and evaporated using a rotary evaporator. The solid white product was recrystallized using ethanol and dried under vacuum at 75 °C for 12 hrs to give 4.89 g of pure monomer (yield $\sim$ 67%).

The relevant spectra for NB-2Me5Cl are given in Figures S15, S16, and S17. In summary, the ^{1}H NMR spectrum of NB-2Me5Cl (500 MHz, $CDCl_3$) exhibits peaks at δ 7.65-7.03 (3H, multiplet), 6.45-6.36 (2H, doublet), 3.65-3.42 (2H, multiplet), 3.05-2.88 (2H, multiplet), 2.50-2.11 (3H, multiplet), and 1.92-1.62 (2H, multiplet). The ^{13}C NMR spectrum (500 MHz, Chloroform-d) reveals peaks at δ 176.52, 137.89, 134.16, 132.15, 129.54, 128.42, 48.33, 47.92, 45.76, 45.17, 43.02, and 17.67. Additionally, the mass spectrum shows peaks at m/z 288.0772 $[M+H]^+$, 305.1041 $[M+NH_4]^+$, 310.0568 $[M+Na]^+$, 575.1464 $[2M+H]^+$, 592.1749 $[2M+NH_4]^+$, 597.1269 $[2M+Na]^+$, and 884.2047 $[3M+Na]^+$, with the HRMS m/z calculated for $C_{16}H_{14}ClNO_2$ $[M+H]^+$ being 288.0713, which matches the observed peak at 288.0772.

S4.7 Synthesis of 2-methyl-5-choro polynorbornene (PNB-2Me5Cl)

The obtained monomer was polymerized using ring-opening metathesis polymerization. In a clean and dry 500 ml single neck round bottom flask, 2 g of NB-2Me5Cl monomer was dissolved in 22 ml DCM under argon at room temperature. Grubbs 2nd generation catalyst (22.6 mg, 0.026 mmol) was dissolved in 4–5 ml of DCM in a vial and added dropwise to the monomer solution. The reaction mixture was stirred for 2 hrs and then quenched using 4–5 ml of ethyl vinyl ether and stirred for 20 more minutes. The solution was then precipitated in cold methanol to get a white polymer. The obtained polymer was further

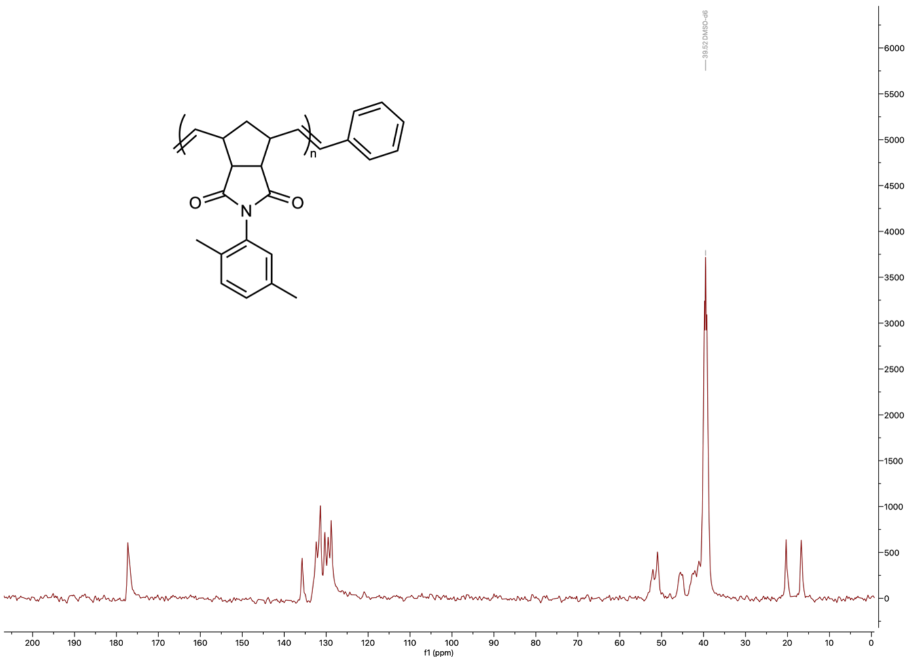

Figure S14: ^{13}C spectrum (300 MHz, DMSO) of PNB-Dimethyl.

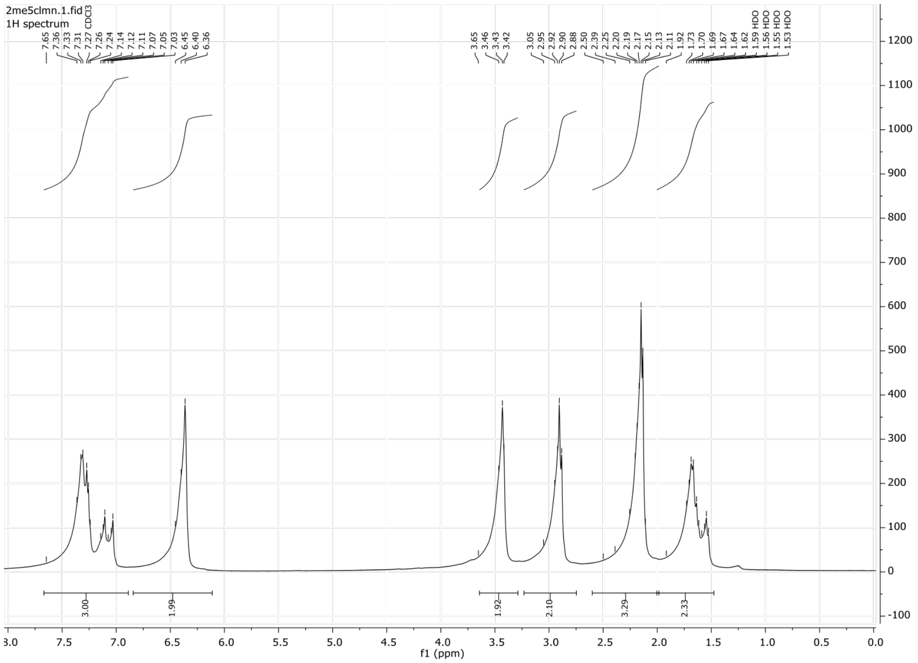

Figure S15: ^{1}H NMR (500 MHz, $CDCl_3$) for NB-2Me5Cl.

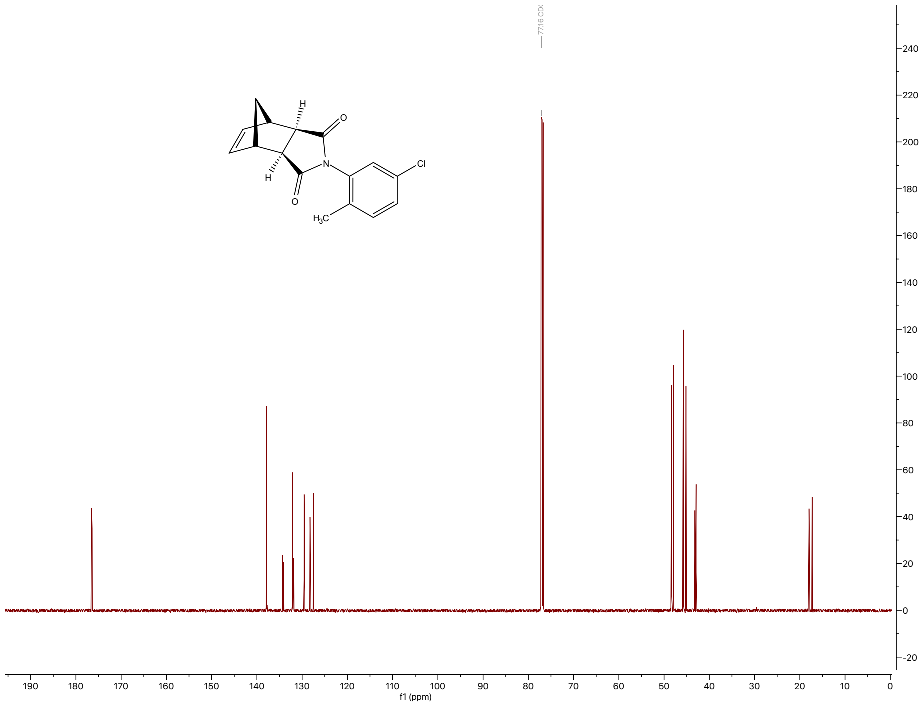

Figure S16: ^{13}C spectrum (500 MHz, $CDCl_3$) of NB-2Me5Cl.

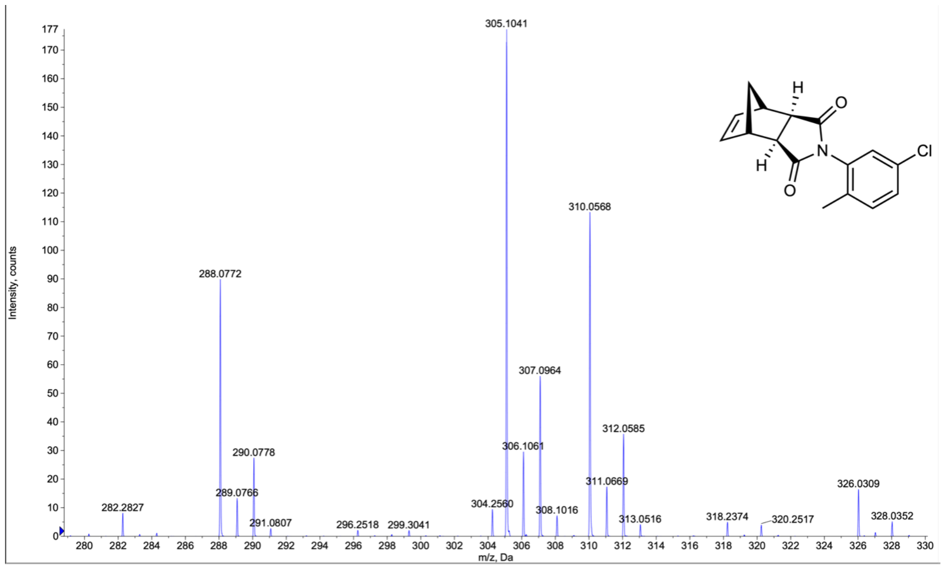

Figure S17: HRMS mass spectrum (positive ion mode) of compound NB-2Me5Cl ($C_{16}H_{14}ClNO_2$).

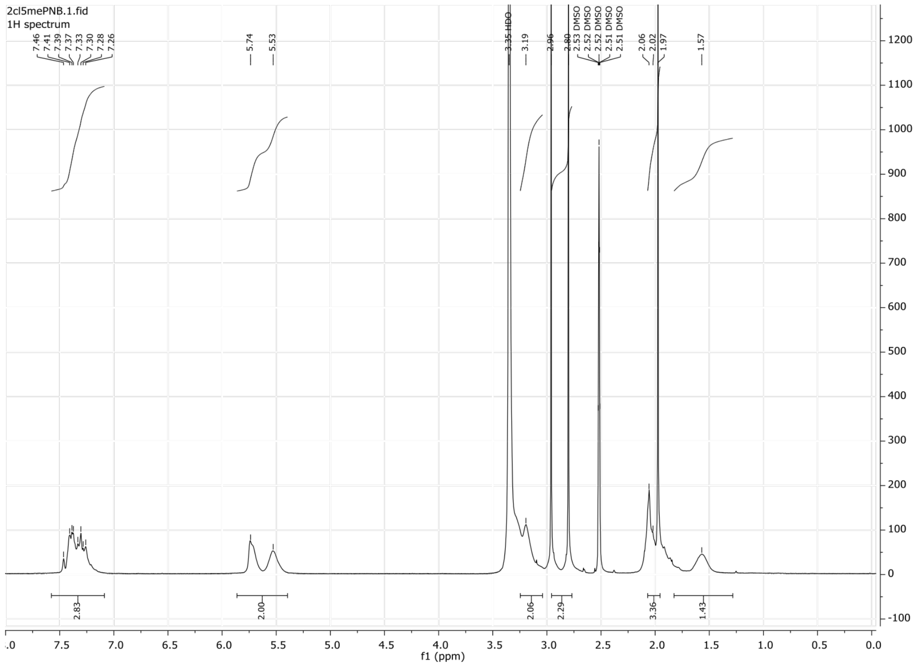

Figure S18: ^{1}H NMR (500 MHz, DMSO) for PNB-2Me5Cl.

purified using Soxhlet extraction with methanol for 48 hrs. The polymer was then dried under vacuum at 75 °C for 24 hrs to give 1.9 g of pure polymer with 95% yield.

The relevant spectra for PNB-2Me5Cl are given in Figures S18 and S19. In summary, the ^{1}H NMR spectrum of PNB-2Me5Cl (500 MHz, DMSO) exhibits peaks at δ 7.46-7.26 (3H, multiplet), 5.74-5.53 (2H, doublet), 3.19 (2H, singlet), 2.96-2.80 (2H, singlet), 2.06-1.97 (3H, multiplet), and 1.57 (2H, singlet). The ^{13}C NMR spectrum (300 MHz, dimethyl sulfoxide-d6) reveals peaks at δ 176.91, 134.97, 132.80, 131.89, 130.24, 128.82, 52.08, 51.11, 45.18, 42.57, and 16.62.

S4.8 Synthesis of 3-chloro-4-methyl norbornene (NB-3Cl4Me)

The monomer was synthesized in a two-step reaction as in Fig. S4. The reaction was carried out in a 500 ml single neck round bottom flask with a magnetic stirrer under an argon atmosphere. For the first step of the reaction, 5 g (30.5 mmol) of cis-5-norbornene-exo-2,3-dicarboxylic anhydride with 30 ml of toluene was added to the flask and stirred. 3-chloro-4-methyl aniline 4.31 g (30.5 mmol) was dissolved in 10 ml toluene and added dropwise to the dispersed solution. The mixture was heated to 40–50 °C for 3 hrs and then cooled down to give a pale-yellow precipitate of amic acid. The precipitate was then filtered and dried under a vacuum to give 8 g (26.2 mmol) of amic acid with $\sim$ 85% yield.

In the second step, 7.5 g amic acid from the first step, $\sim$ 1.22 g sodium acetate, and $\sim$ 8 g acetic anhydride (excess) were added to the flask with a magnetic stirrer, and the mixture was then heated to 60–70 °C for 8 hrs. The reaction mixture was cooled down to room temperature, and a solid white precipitate crashed out. The precipitate was filtered under the vacuum and then washed several times with water by extraction using dichloromethane (DCM). The collected organic layer was then collected and evaporated using a rotary evaporator. The solid white product was recrystallized using ethanol and dried under vacuum at 75 °C for 12 hrs to give 3.2 g of pure monomer (yield $\sim$ 55%).

The relevant spectra for NB-3Cl4Me are given in Figures S20, S21, and S22. In summary, the ^{1}H NMR spectrum of NB-3Cl4Me (500 MHz, $CDCl_3$) exhibits peaks at δ 7.37-7.33 (2H, multiplet), 7.14-7.11 (1H, multiplet), 6.39 (2H, singlet), 3.45-3.44 (2H, singlet), 2.89 (2H, singlet), 2.44 (3H, singlet), and 1.68-1.49 (2H, multiplet). The ^{13}C NMR spectrum (500 MHz, Chloroform-d) reveals peaks at δ 176.69, 137.92, 136.80, 134.60, 131.13, 130.31, 126.84, 124.49, 47.66, 45.65, 43.04, and 19.70. Additionally, the mass spectrum shows peaks at m/z 288.0772 $[M+H]^+$, 305.1041 $[M+NH_4]^+$, 310.0635 $[M+Na]^+$, 597.1269 $[2M+Na]^+$, and 884.2047 $[3M+Na]^+$, with the HRMS m/z calculated for $C_{16}H_{14}ClNO_2$ $[M+H]^+$ being 288.0713, which matches the observed peak at 288.0772.

S4.9 Synthesis of 3-chloro-4-methyl polynorbornene (PNB-3Cl4Me)

The obtained monomer was polymerized using ring-opening metathesis polymerization. In a clean and dry 500 ml single neck round bottom flask, 2 g of NB-3Cl4Me monomer was dissolved in 22 ml DCM under argon at room temperature. Grubbs 2nd generation catalyst (22 mg, .026 mmol) was dissolved in 4–5 ml of DCM in a vial and added dropwise to the monomer solution. The reaction mixture was stirred for 2 hrs and then quenched using 4–5 ml of ethyl vinyl ether and stirred for 20 more minutes. The solution was then precipitated in cold methanol to get a white polymer. The obtained polymer was further purified using Soxhlet extraction with methanol for 48 hrs. The polymer was then dried under vacuum at 75 °C for 24 hrs to give 1.8 g of pure polymer with 90% yield.

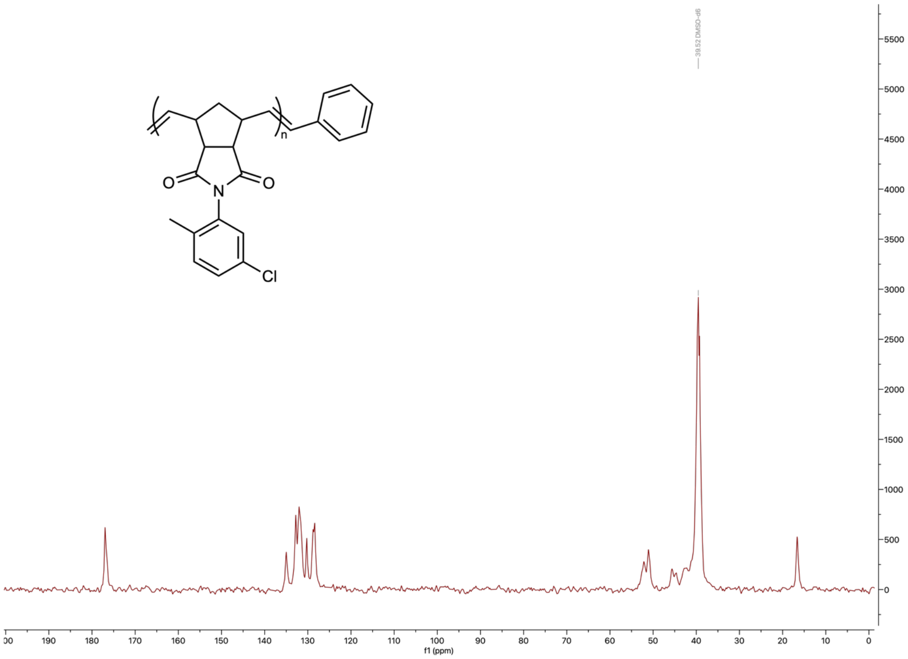

Figure S19: ^{13}C spectrum (300 MHz, DMSO) of PNB-2Me5Cl.

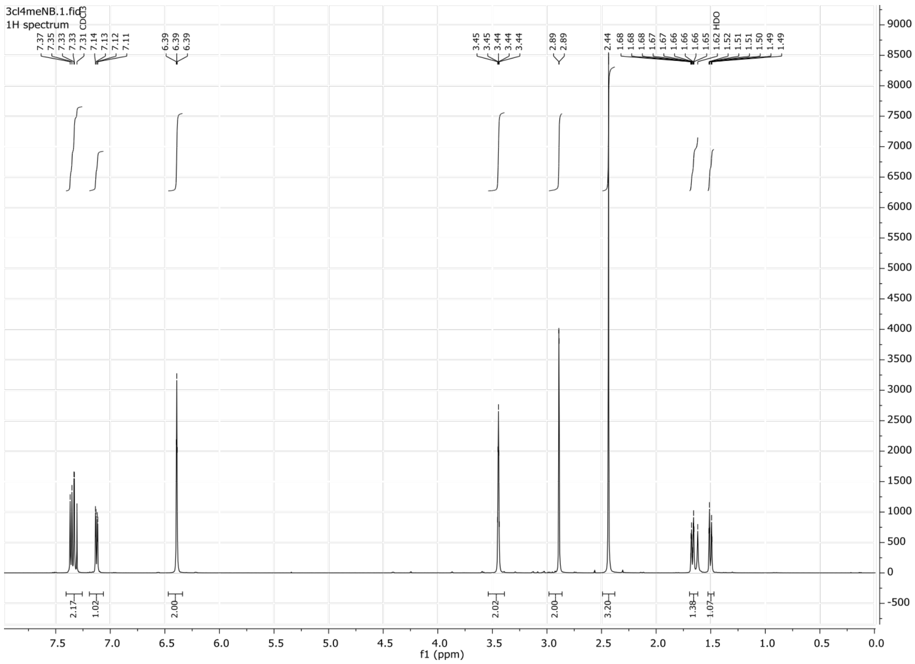

Figure S20: ^{1}H NMR (500 MHz, $CDCl_3$) for NB-3Cl4Me.

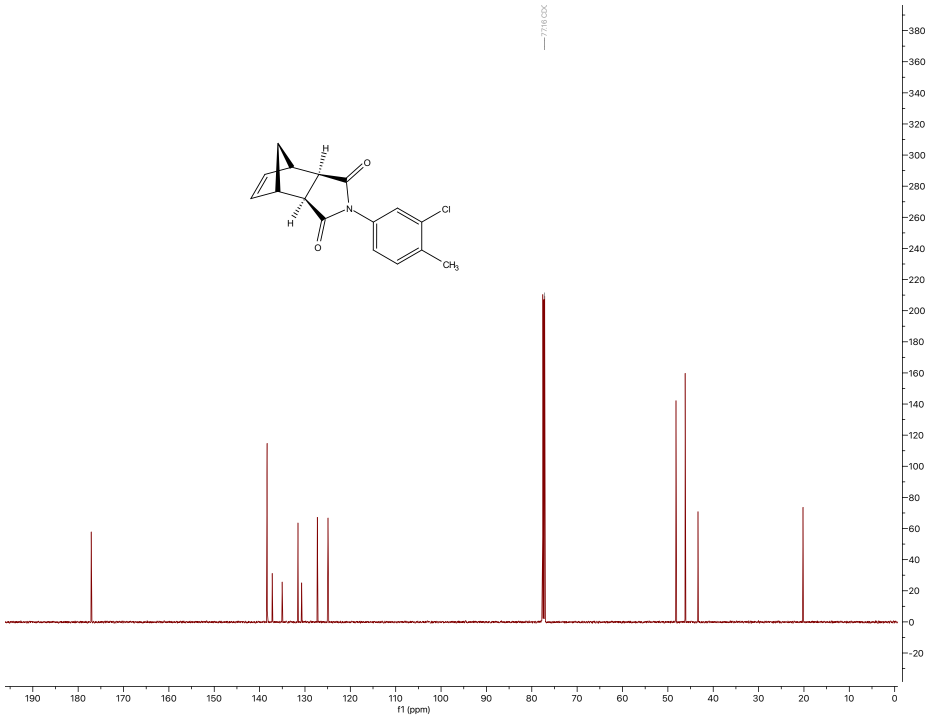

Figure S21: ^{13}C spectrum (500 MHz, $CDCl_3$) of NB-3Cl4Me.

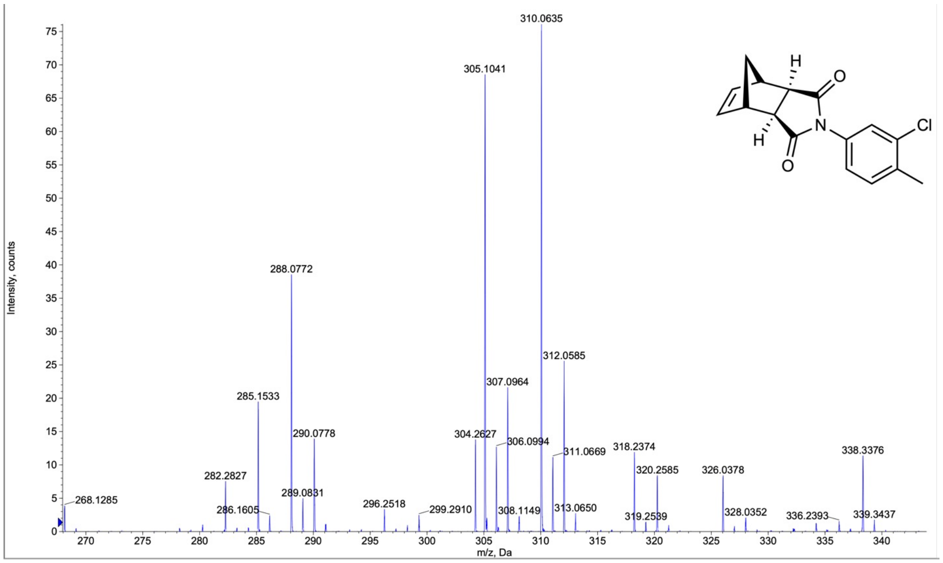

Figure S22: HRMS mass spectrum (positive ion mode) of compound NB-3Cl4Me ($C_{16}H_{14}ClNO_2$).

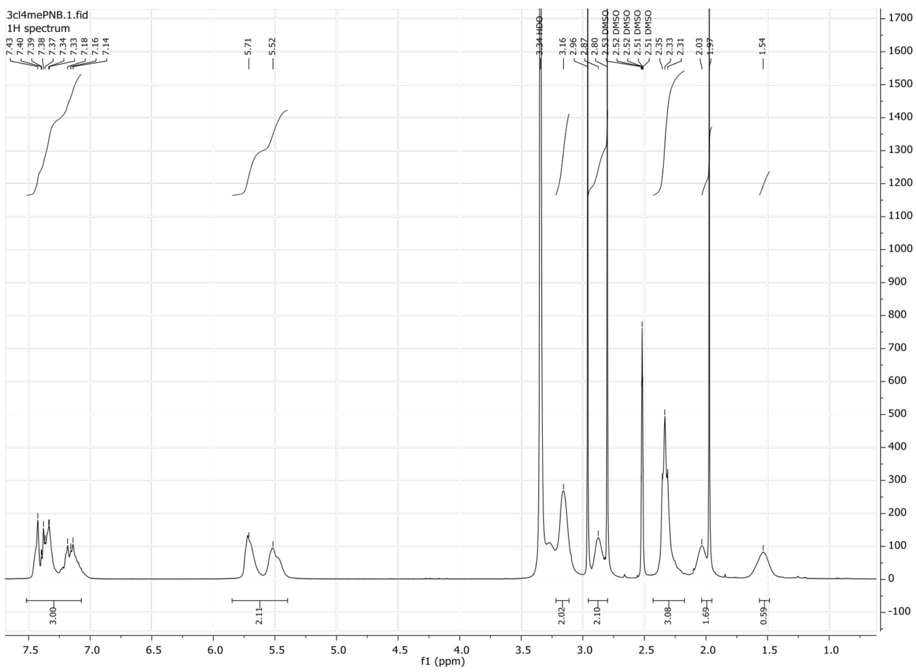

Figure S23: ^{1}H NMR (500 MHz, DMSO) for PNB-3Cl4Me.

The relevant spectra for PNB-3Cl4Me are given in Figures S23 and S24. In summary, the ^{1}H NMR spectrum of PNB-3Cl4Me (500 MHz, DMSO) exhibits peaks at δ 7.43-7.14 (3H, multiplet), 5.71-5.52 (2H, doublet), 3.16 (2H, singlet), 2.96-2.80 (2H, singlet), 2.35-2.31 (3H, doublet), and 2.03-1.54 (2H, singlet). The ^{13}C NMR spectrum (300 MHz, dimethyl sulfoxide-d6) reveals peaks at δ 177.30, 135.46, 132.85, 131.40, 131.09, 127.38, 125.89, 51.44, 44.89, 42.72, 21.46, and 19.29.

S5 Casting films

All freestanding polymer films were processed using the solution casting method with the help of a Doctor Blade film coater. Tetrahydrofuran (THF) or Dichloromethane (DCM) was used as the solvent to make a 6–10% concentrated solution of the polymer. The solution was then filtered using a 0.45 microfilter to remove undissolved materials. Then, after producing a film with the Doctor Blade, the film was placed on a glass substrate at 23 °C for half an hour for DCM and 2 hours for THF. The film was left to dry overnight. Then the film was removed from the glass substrate using deionized water. The polymer film was then dried in a vacuum oven at 60–70 °C for a day to remove solvent residue.

S6 Material characterization

S6.1 Gel permeation chromatography (GPC)

A Waters GPC system was used with dimethylacetamide (DMAc) as a mobile system and polystyrene (PS) as a standard with a refractive index (RI) detector to calculate the molecular weight of the polymers. The GPC was calibrated against PS of varying molecular weights (1090000, 392000, 189000, 32660, 9100, 6100, 2300 Da). The molecular weight and dispersity (Đ) of all synthesized polymers is shown in Table S2. The GPC traces are shown in Fig. S25.

	M_n	M_w	Đ
PONB-2Me5Cl	1.0620E+05	3.7119E+05	3.50
PNB-Dimethyl	2.4927E+05	9.0679E+05	3.64
PNB-2Me5Cl	4.2505E+05	1.2691E+06	2.99
PNB-3Cl4Me	3.1618E+05	1.2103E+06	3.83

Table S2: Molecular weight data of all synthesized polymers.

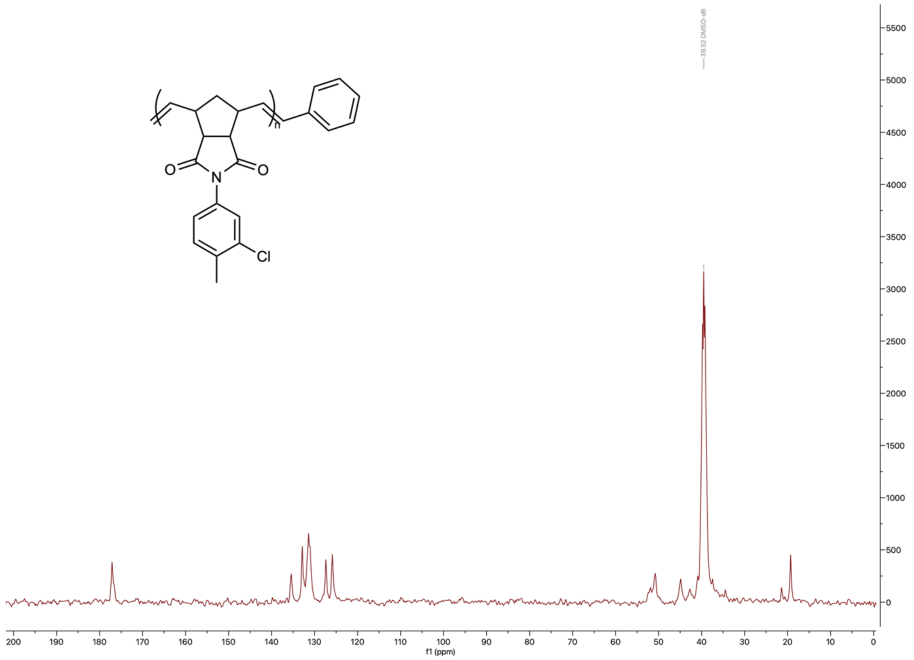

Figure S24: ^{13}C spectrum (300 MHz, DMSO) of PNB-3Cl4Me.

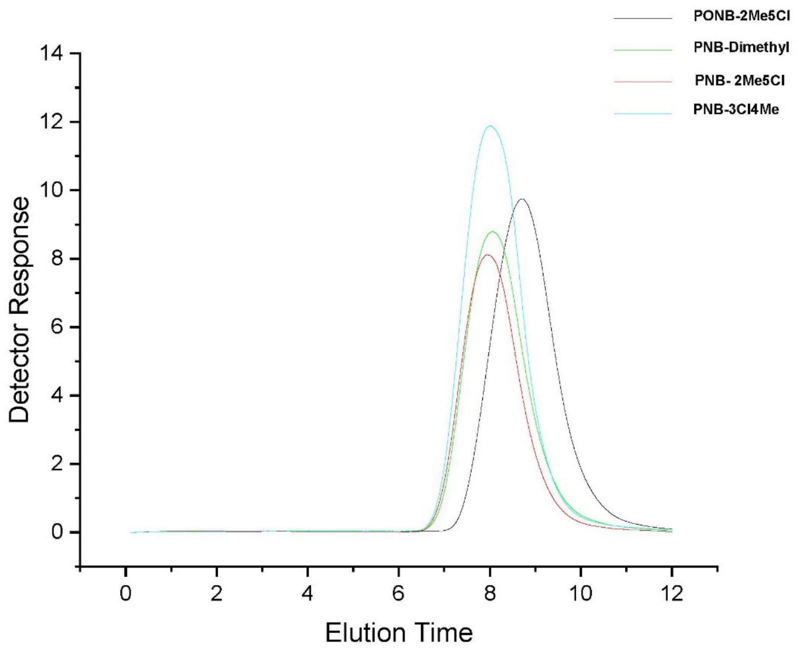

Figure S25: GPC traces of all synthesized polymers.

S7 Thermal characterization

S7.1 Thermal Gravimetric Analysis (TGA)

TGA was used to study the thermal degradation of polymers under inert atmosphere. The degradation study under nitrogen shows that the 5% degradation temperature (T_d) of PONB-2Me5Cl and PNB-Dimethyl polymers were higher than 400 °C, which indicates high thermal stability. Meanwhile, the 10% T_d measurements of the other two polymers, PNB-2Me5Cl and PNB-3Cl4Me, are around 409 °C and 422 °C respectively. Although this reflects a lower thermal stability than that of other predicted polymers, the stability is still relatively high. These results are shown in Fig. S26.

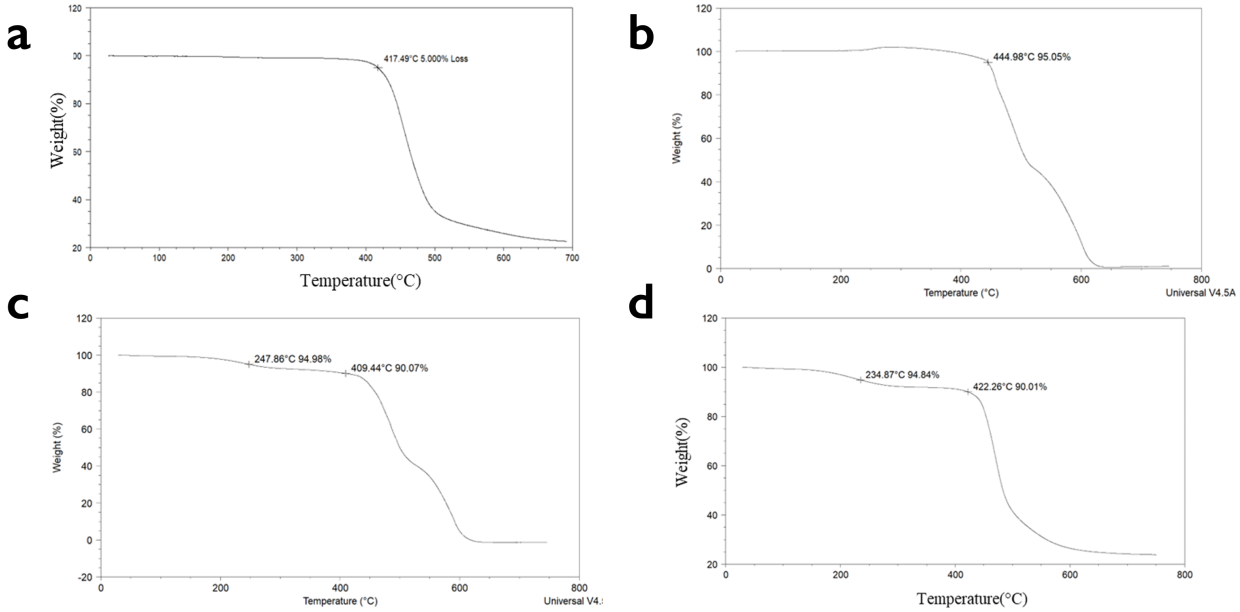

Figure S26: Thermal Gravimetric Analysis of a) PONB-2Me5Cl, b) PNB-Dimethyl, c) PNB-2Me5Cl, d) PNB-3Cl4Me.

S7.2 Differential Scanning Calorimetry (DSC)

DSC is used to observe the T_g of the polymers. The T_g of PONB-2Me5Cl, PNB-Dimethyl, PNB-2Me5Cl, and PNB-3Cl4Me was observed, respectively, around 232 °C, 232 °C, 243 °C and 220 °C as shown in Fig. S27. It can be clearly seen that all polymers have T_g greater than 200 °C, the reason being the presence of a rigid bicyclic ring in the polymer backbone. We can also see, from the case of PONB-2Me5Cl and PNB-2Me5Cl, that the change in the bridgehead atom from oxygen to carbon in the bicyclic norbornene ring causes an increase in the T_g of the polymer. We also observed that when the position of the pendant group is ortho and meta, the T_g of the polymer is higher than when the position of the pendant group is meta and para. Restricted rotation at the ortho position due to the presence of imide causes an increase in T_g at the ortho position compared to the para and meta positions. The high T_g is an essential criterion for dielectric materials to function under extreme thermal as well as electric field conditions.

S8 Electronic characterization

The charge-discharge curves for the polymers studied in this work are shown in Fig. S28 and Fig. S29. The displacement-electric field (D-E) loops are shown in Fig. 3a of the main body and in Fig. S30. The energy density as a function of temperature for all polymers is shown in Fig.

S8.1 Band gap measurement

PerkinElmer's Lambda 1050 UV/VIS/NIR spectrometer was used to measure the electronic band gap E_g of the PONB-2Me5Cl, PNB-Dimethyl, PNB-2Me5Cl, PNB-3Cl4Me polymers. Samples were prepared by making a dry, transparent, freestanding thin film of polymers. Samples were scanned over the wavelength range of 200–800 nm. The onset of absorbance wavelength λ_{onset} was used for the band gap calculations:

$$E_g = \frac{1240}{\lambda_{\mathrm{onset}}}$$

The λ_{onset} for PONB-2Me5Cl, PNB-Dimethyl, PNB-2Me5Cl, and PNB-3Cl4Me were observed at around 282 nm, 285 nm, 287 nm, and 290 nm, respectively, as shown in Fig. S32. These λ_{onset} correspond to a band gap of 4.39 eV, 4.34 eV, 4.32 eV, and 4.27 eV, respectively.

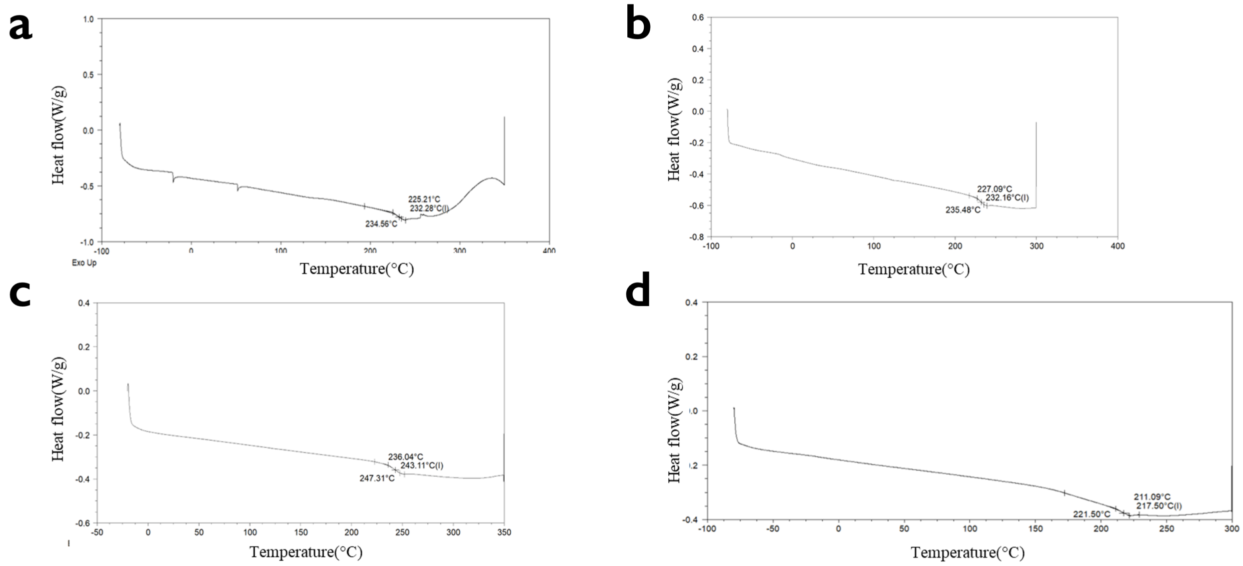

Figure S27: Differential Scanning Calorimetry of a) PONB-2Me5Cl, b) PNB-Dimethyl, c) PNB-2Me5Cl, d) PNB-3Cl4Me.

For a polymer to have good insulating properties, the band gap should be more than 3 eV. The polynorbornenes studied here show a better band gap (greater than 4 eV) compared to other commercially available insulating polymers that have glass transition temperatures (T_g) greater than 100 °C. The reason is that common high T_g polymers have aromatic benzene rings present in their backbone, which causes conjugation and π-π stacking the backbone, leading to a low band gap. However, in our selected polynorbornenes, the presence of a bicyclic ring in the backbone breaks this design constraint of the presence of conjugation in the polymer backbone without compromising the T_g of the polymer, making them good candidates for high-temperature polymer dielectrics.

S9 Proposed polyimide synthesis

Four green polyimides for high temperature, high energy density dielectrics are proposed in Fig. 5 of the main body. Each polyimide may be synthesized using a dianhydride and a diamine. The dianhydrides may be purchased directly while the diamines can be synthesized from starting materials in one or two steps, according to CAS SciFinder®.

S10 Solubility model

Before training a production solubility model using the whole data set, an identical model was trained on two-thirds of the polymer-solvent pairs and tested on the remaining third.
Five-fold cross-validation was used. Eighty percent of the training data (the "fitting data") was used to fit the model over 1000 epochs. The remaining twenty percent (the "validation data") was used to select the best model out of the 1000 epochs. Figure S33 displays, as a function of epoch number, the mean and standard deviation over the five folds for accuracy on fitting data, F1 score on fitting data, accuracy on validation data, and F1 score on validation data. The confusion matrix for the 8895 unseen polymer-solvent pairs is shown in Figure S34.

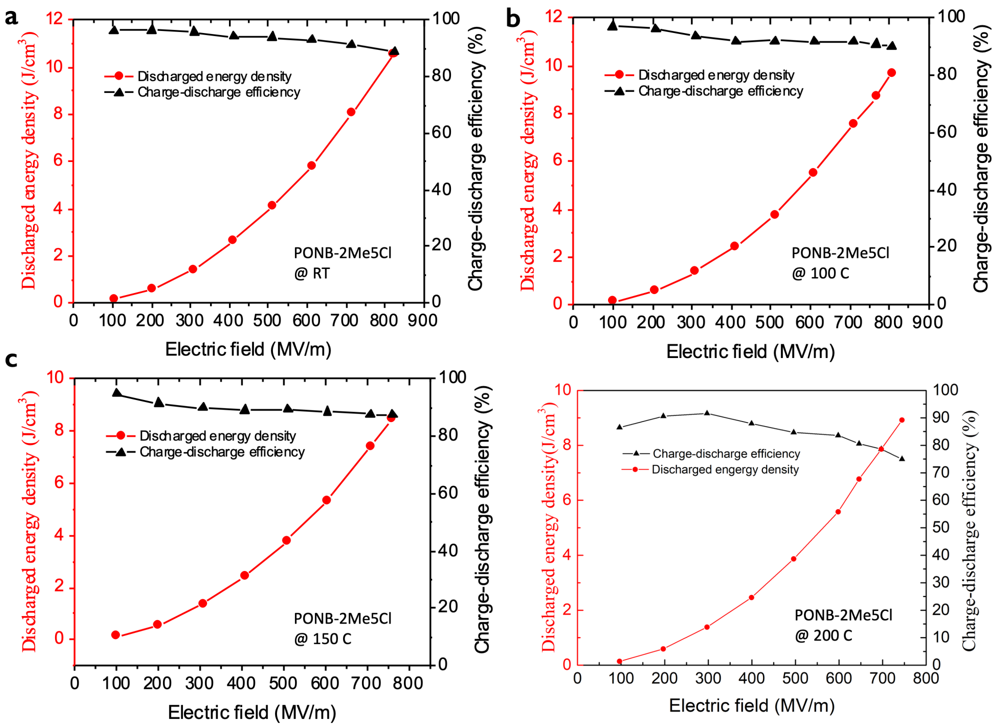

Figure S28: Discharged energy density and charge-discharge efficiency of PONB-2Me5Cl at a) room temperature (RT), b) 100 °C, c) 150 °C, d) 200 °C.

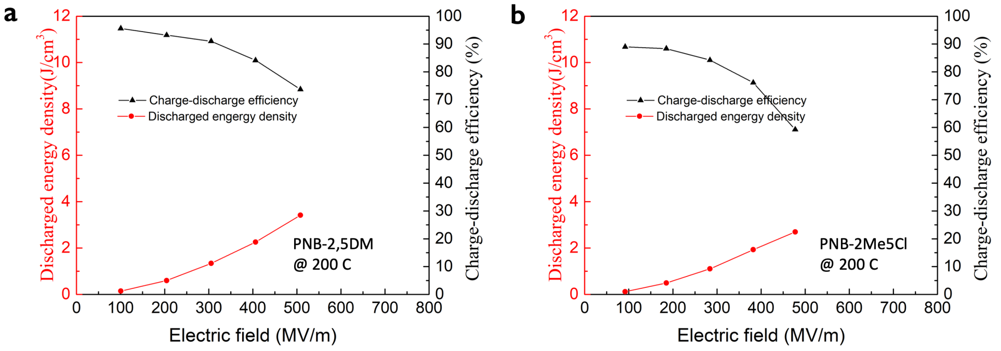

Figure S29: Discharged energy density and charge-discharge efficiency at 200 °C for a) PNB-2,5DM and b) PNB-2Me5Cl.

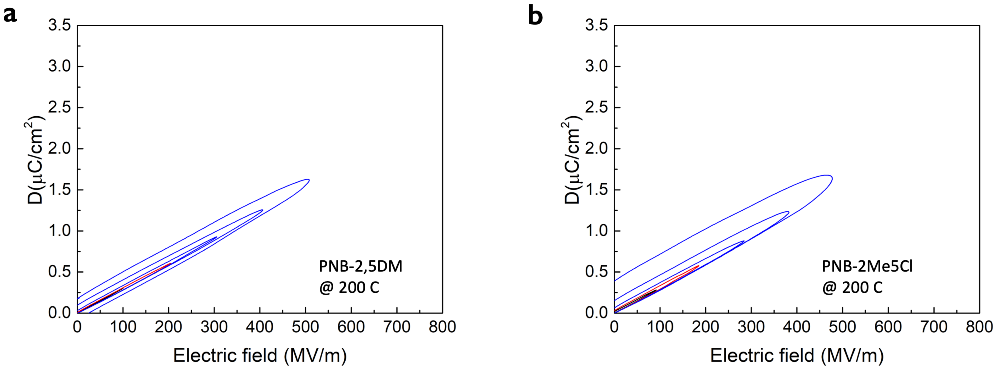

Figure S30: D-E loops at 200 °C for a) PNB-2,5DM and b) PNB-2Me5Cl.

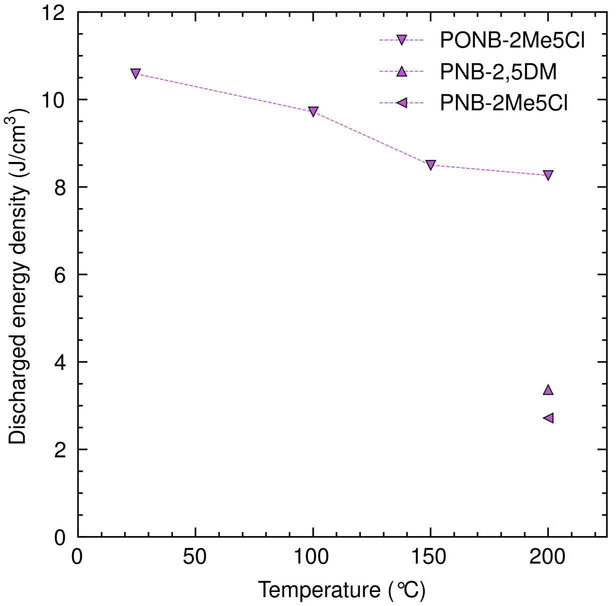

Figure S31: Discharged energy density vs. temperature for the polymers studied in this work.

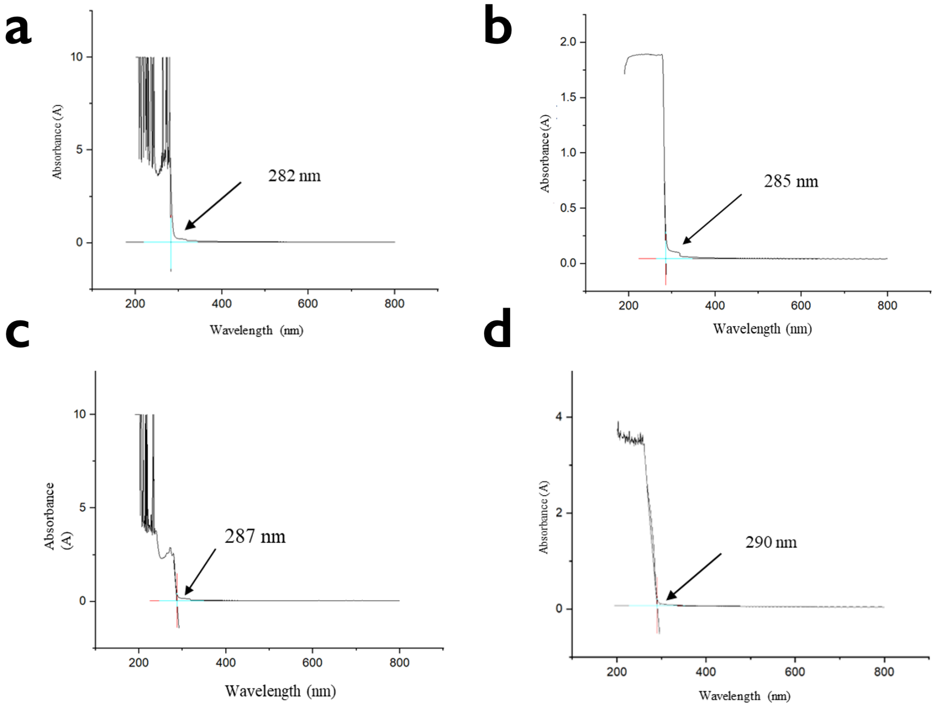

Figure S32: Band gap determined using UV-vis spectroscopy a) PONB-2Me5Cl, b) PNB-Dimethyl, c) PNB-2Me5Cl, d) PNB-3Cl4Me

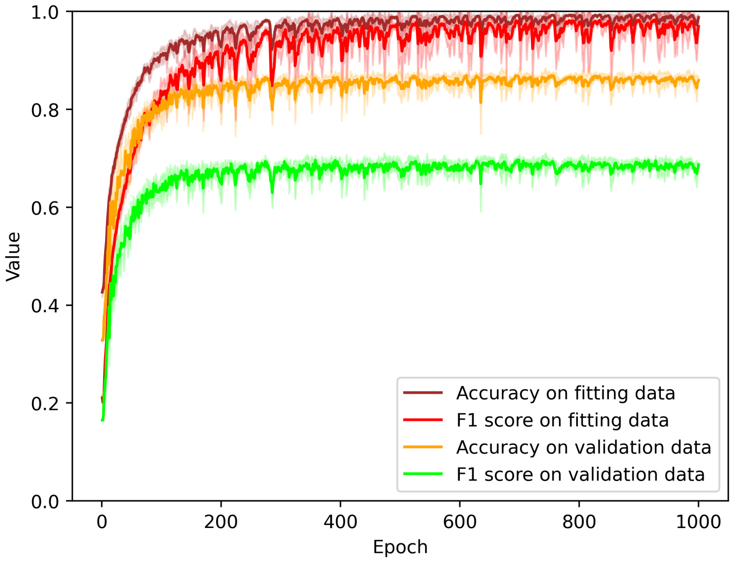

Figure S33: Solubility model metrics as a function of epoch.

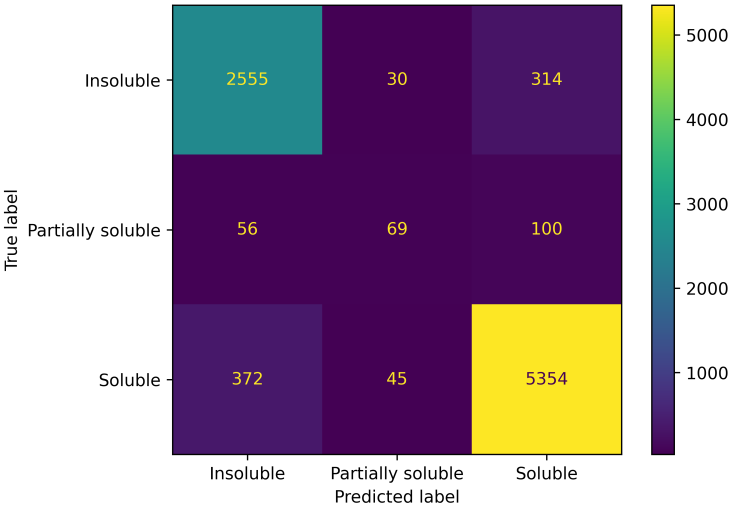

Figure S34: Confusion matrix for unseen data using the solubility model.

References

[1] R. Gurnani, C. Kuenneth, A. Toland, and R. Ramprasad, "Polymer Informatics at Scale with Multitask Graph Neural Networks," *Chemistry of Materials*, vol. 35, no. 4, pp. 1560–1567, 2 2023. [Online]. Available: https://doi.org/10.1021/acs.chemmater.2c02991

[2] A. A. Santiago, J. Vargas, S. Fomine, R. Gaviño, and M. A. Tlenkopatchev, "Polynorbornene with pentafluorophenyl imide side chain groups: Synthesis and sulfonation," *Journal of Polymer Science Part A: Polymer Chemistry*, vol. 48, no. 13, pp. 2925–2933, 7 2010. [Online]. Available: https://onlinelibrary.wiley.com/doi/full/10.1002/pola.24073 https://onlinelibrary.wiley.com/doi/abs/10.1002/pola.24073 https://onlinelibrary.wiley.com/doi/10.1002/pola.24073
